# Evidence that hematopoietic stem cells in human umbilical cord blood is infectable by dengue virus: proposing a vertical transmission candidate

**DOI:** 10.1016/j.heliyon.2021.e06785

**Published:** 2021-04-20

**Authors:** Amrita Vats, Tzu-Chuan Ho, Irwin Puc, Yi-Ju Chen, Chiung-Hsin Chang, Yu-Wen Chien, Guey-Chuen Perng

**Affiliations:** a Institute of Basic Medical Sciences, College of Medicine, National Cheng Kung University, Tainan, Taiwan; b Department of Microbiology and Immunology, College of Medicine, National Cheng Kung University, Tainan, Taiwan; c Department of Obstetrics and Gynecology, College of Medicine, National Cheng Kung University, Tainan, Taiwan; d Department of Public Health, College of Medicine, National Cheng Kung University, Tainan, Taiwan; e Department of Occupational and Environmental Medicine, National Cheng Kung University Hospital, College of Medicine, National Cheng Kung University, Tainan, Taiwan

**Keywords:** Dengue virus, Umbilical cord blood, CD133^+^, CD34^+^, Hematopoietic stem cells

## Abstract

**Background:**

Recent studies have shown that dengue virus (DENV) can efficiently infect bone marrow hematopoietic stem cells (HSCs) as well as the placenta of pregnant women. Although mother-to-infant vertical transmission of DENV through the placenta has been well documented, the evidence of cell-associated vertical transmission is still unknown. Whether DENV can infect umbilical cord blood (UCB) cells before reaching the fetus remains to be explored. Here, we proposed that human UCB cells were permissive to the DENV infection and DENV infected CD133+ and CD34+ HSCs are reservoir of the virus that could be reactivated upon re-culturing in suitable cells.

**Methods:**

Human UCB cells were freshly obtained and subjected to DENV infection. Multicolor flow cytometry (MFCM) was used to demonstrate the phenotypes of the infected HSC populations. Immunofluorescence analysis (IFA) and T-distributed Stochastic Neighbor Embedding (t-SNE) were used to show the association of the DENV antigen, non-structural protein1 (NS1) with HSCs.

**Key findings:**

UCB cells were highly permissive to DENV infection. DENV altered the phenotype of the infected HSC population, increased the expression of HSCs, and affected the balance of transcription factors (TFs, GATA1/2/3). IFA revealed the association of the DENV antigen, non-structural protein1 (NS1), with CD34^+^ and CD133^+^ cells. T-distributed Stochastic Neighbor Embedding (t-SNE) analysis revealed heterogeneity in the distribution of CD133^+^NS1^+^, and CD34^+^ NS1^+^ cells. DENV particles were recovered from CD133^+^ and CD34^+^ cells even when virus production in the supernatant was negligible.

**Significance:**

We predict that infection of CD133+ and CD34+ cells in the UCB serve as reservoirs for the amplification of DENV in UCB prior to the virus reaching the fetus and facilitate vertical transmission.

## 1 Introduction

Dengue is a serious global public health problem in both tropical and sub-tropical areas. According to the World Health Organization (WHO), 3.9 billion people in 128 countries are currently at risk of dengue infection, and most cases are asymptomatic [1, 2]. The dengue virus (DENV), an arbovirus of the family Flaviviridae, is frequently transmitted to humans by *Aedes spp.* Mosquitoes [3, 4]. Cases of dengue have also been documented to be infected through unconventional routes, such as blood transfusion, bone marrow, stem cells, and organ transplantations [5, 6]. However, vertical transmission of DENV from mother to fetus has received relatively little attention [7, 8, 9]. In one prospective study, 5.8% of newborns showed warning signs of congenital infection and vertical transmission rates were estimated to be as high as 18.5% [10]. In a Brazilian case study, approximately 30% of pregnant women with symptomatic dengue had at least one clinical or biological warning sign, and they were 3.4 times more likely to develop severe dengue [11]. Moreover, maternal dengue carries the risk of adverse fetal outcomes, such as miscarriage, stillbirth, preterm birth, and low birth weight and subclinical or inapparent infection in young children [12, 13]. One to five percent of infants hospitalized due to severe dengue have dengue-immune mothers [14, 15].

DENV neutralizing antibody is commonly present in mothers of infants with primary dengue in endemic countries [16, 17]. Seropositivity rate was found to be 35.8% in Malaysia and as high as 94.7% in Thailand [18, 19]. Maternally derived, neutralizing anti-DENV antibodies are postulated to confer immunity to dengue during the first months of life [20]. The prime cause of fetal and neonatal morbidity and mortality in DENV infection during pregnancy is insufficient maternally derived IgG neutralizing antibodies. The incremental decay of the IgG antibody generates a window period that possesses sub-neutralizing levels of antibody, which effectively enhances DENV infection in Fc receptor-bearing host cells and uncontrollable viremia, implying the corollary of antibody-dependent enhancement of DENV infectivity [19, 20]. However, it is still mysterious whether sub-neutralizing levels of maternally derived anti-DENV IgG increases fetal DENV infection risk. Ample evidence suggests that increasing IgG catabolism, due to high competition and mutation among IgG, causes loss of binding and interaction to FcRn receptors, which reduces the transfer of maternal IgG antibodies to neonates [21, 22]. The ambiguous nature of maternally derived neutralizing antibody makes it much more difficult to define its roles in DENV transmission to the fetus.

A growing body of evidence indicates that bone marrow hematopoietic stem and progenitor cells are permissive to DENV infection and can efficiently infect hematopoietic stem cells, leading marrow suppression [23, 24, 25, 26]. Vertical transmission of DENV poses a risk of fetal death, because of thrombocytopenia in the newborn that entails platelet transfusion [27, 28]. The complex process of platelet production starts with hematopoietic stem cell (HSC) differentiation and megakaryocyte (MK) lineage commitment, which is followed by MK maturation and platelet release [29, 30]. Congenital DENV infection usually occurs during late pregnancy and is caused by direct transfer of the virus across the maternal placenta through the umbilical cord blood (UCB), thus resulting in neonatal infection [31, 32]. Cord blood leucocytes have been shown to support DENV2 replication in vitro [33]. DENV has been discovered in the placenta, UCB, and serum of fetuses in pregnant women [34, 35]. UCB, a critical bridge and carrier that transfers nourishment from the mother to the fetus, has been found to contain maternal cell contaminants with a substantial amount of hematopoietic stem and progenitor cells, including CD34 cells [36, 37]. Additionally, these cells may be capable of producing memory B cells or maternal antibodies in fetus [38, 39].

Intrigued by these findings, we attempted to assess the role of a hematopoietic component in UCB in vertical transmission and its specificity to DENV protein. Hence, we investigated the feasibility of targeting primitive hematopoietic cells CD34 and CD133 and their association with DENV NS1 protein. CD34 and CD133 are cell surface glycoproteins found specifically in undifferentiated cells with approximately 0.02–1.43%, and 0.1–0.4% mononuclear cells of UCB are CD34^+^ and CD133^+^, respectively [40, 41]. The purpose of this study was to assess the potency of CD34^+^ CD133^+^ HSCs in UCB to be infected by DENV and the effects of DENV infection to the expression of hematopoietic GATA factors to demonstrate the functional abnormalities during hematopoietic differentiation in DENV-infected UCB. GATAs are essential regulators in the development of hematopoietic cell lineage and the molecular pathogenesis of diseases [42]. A series of publications, mainly registered case reports, highlighting the clinical history of maternal dengue during pregnancy with no apparent explanation accounting for the significant carrier of DENV from mother to fetus and cellular level mechanism of vertical transmission [43, 44, 45]. We therefore hypothesized that stem cells in UCB might serve as an amplification reservoir for DENV during vertical transmission from the placenta to the neonate. We have therefore established an in vitro experimental model to document that DENV indeed efficiently infects human umbilical cord blood cells (HUCB) and serves as a potential carrier of DENV.

## 2 Methods

### 2.1 Human umbilical cord blood collection and preparation of the UCB for DENV infection

Pregnant women with written informed consent participated in this study, following the protocol (IRB, A-ER-103-184) approved by the Institutional Review Board of National Cheng Kung University Hospital. Total UCB cells were counted after initial treatment to remove red blood cells by RBC lysis buffer (Qiagen Inc, USA). 2 × 10^7^ cells/ml were suspended in RPMI medium (Hyclone, USA) supplemented with 10% FBS (Gibco, Ireland) and 1 × 10^7^ cells/ml were infected with DENV-2 (16881 strain) with 1 MOI (Multiplicity of infection). In parallel, a control tube of uninfected UCB cells (1 × 10^7^ cells/ml) was prepared. Supernatants and cells were harvested at specific time points of post-infection (PI) days (D0, D1, D2, D3, D5, D7, D10, and D14) to quantify the viral load from DENV-infected UCB supernatant using plaque assay. The detail descriptions of the protocols were provided in Supplementary Data.

### 2.2 Immunophenotyping and multicolor flow cytometry analysis (MFCM)

The cells harvested at indicated time points were subjected to MFCM by staining with fluorescence-conjugated antibodies for phenotypic analysis of hematopoietic stem and progenitor cells (HSPC) in infected and uninfected cells. All staining was performed in PBS containing 1% BSA. Mouse anti-human surface marker antibodies, specific for the HSC, sub-populations of myeloid cell lineage and GATA TFs with their respective isotype controls (BD Biosciences, Research and Design), listed in the Supplementary Table S6A-B. The cells were acquired using LSR Fortessa (BD Bioscience) for MFCM analysis. To analyze and confirm the association of NS1 expression with CD133 or CD34 cells in DENV-infected UCBs, IFA was performed from D1 to D14. Fluorescent images were captured in an Inverted Confocal Microscope FV-1000 (Olympus). The detail methods were described in Supplementary Data.

### 2.3 Sorting of CD133^+^ and CD34^+^ cells and co-culture with Vero cells

CD133 and CD34 markers of HSCs were isolated from UCB cells by magnetic cell sorting (Miltenyi Biotec) a cell sorter (MoFlo XDP cell sorter, Beckman Coulter). A total of 1× 10^8^ cord blood cells was processed to isolate pure populations of the positive and negative fractions of CD34 and CD133 cells, respectively. The sorted CD34^+^ and CD133^+^ cells were then infected with DENV at MOI = 1 and cultured in 10% RPMI to monitor the virus production at the indicated time points from D0 to D14. In addition, CD34^+^ and CD133^+^ cells with negligible virus production in supernatants were subsequently co-cultured with Vero cells to recover the infectious virus (Figure 7B). 1×10^5^ Vero cells per well were seeded in 24-well microplates one day before the harvested time point to allow the formation of a monolayer. CD133^+^ and CD34^+^cells reconstituted with 500 μl of 2% DMEM were co-incubated with Vero cells (5% CO_2_, 37 °C) for 7 days to observe cytopathic changes. Two hundred microliter of the supernatant was collected daily from day 2 to day 7 post co-cultures, and each well was replenished with an equal amount of 2% FBS DMEM. The co-cultured supernatant was subjected to plaque assay.

### 2.4 Temporal heterogeneity of CD133, CD34, and NS1 using t-SNE

To observe the spatial and temporal changes of stem cell populations, double-positive NS1^+^CD34^+^ and NS1^+^CD133^+^ cells from each of the 7 donors were clustered together to generate a single concatenate file using coloring parameters from Flow Jo version 10. This technique enabled us to create a t-SNE distribution map of different time points during DENV infection in UCBs. It was ensured that debris was excluded during the gating of the live cells based on FSC/SSC plot. The density of different levels of CD34^+^NS1^+^ and CD133^+^NS1^+^ expressed over time were color-coded. The parameters utilized to generate the t-SNE map were as follows: down sample = 50,000 cell events, iteration = 1000 and perplexity = 100. Next, we systematically assess the expression level of CD133^+^ on CD34^+^NS1^+^ and CD34^+^ on CD133^+^NS1^+^ cell density plot using t-SNE analysis. The detail descriptions of the protocol were provided in Supplementary Data.

## 3 Results

### 3.1 Human UCBs were infectable by DENV, resulting in an increase of HSC population and a decrease in progenitor cells

**Figure 1:**
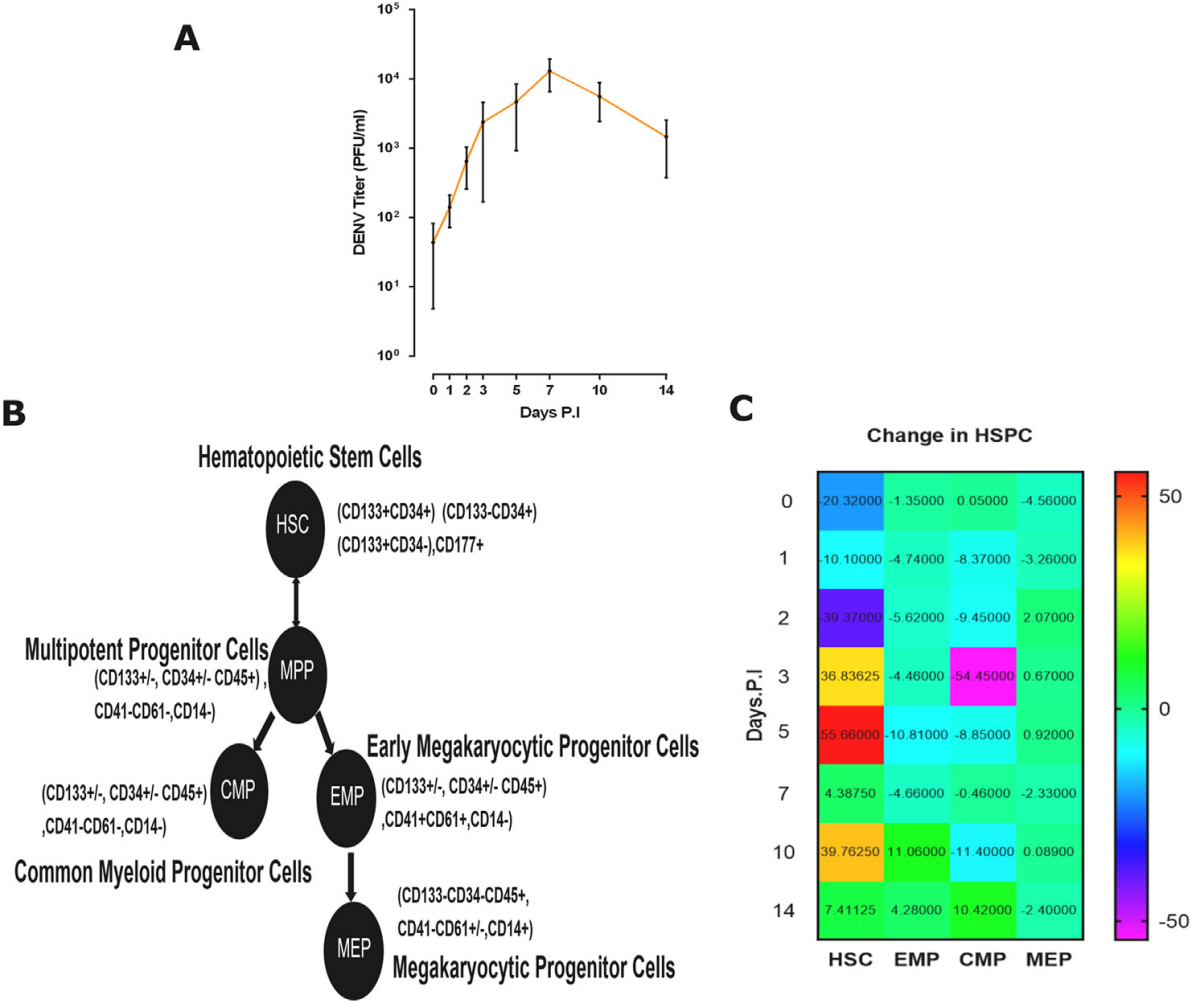
Human UCBs were infectable by DENV, resulting in an increase of HSC population and a decrease in progenitor cells (A) Average viral titers in supernatant collected from the cultured medium of UCB after dengue viral infection. Data represent 7 separates infected UCB supernatant n = 7. Error bar represent standard error of the mean ± SEM of (D0) 69.413, (D14) 1086.042. (B) Classification of hematopoietic stem cells. (C) Change of different types of HSPC populations in DENV-infected UCB (N = 7). CD markers were used as criteria to separate the cells into different cell populations including HSC, CMP, MEP, and EMP. Please also referred to Supplementary Figure S1 and Supplementary Figure S2.

We first investigated the permissivity of UCB to DENV by determining whether DENV amplification took place in UCB. The average viral titer of DENV-infected UCBs supernatant demonstrated that UCB could be efficiently infected by the DENV with a peak titer on day 7 (Figure 1A), although a variation in the individual donors was observed, denoted in Supplementary Figure S1 and Supplementary Table S1. We then compared the distribution of the surface markers-defined phenotypes of HSPC from the cells collected from DENV-infected UCB. Based on the established markers of HSPC (Figure 1B) [46], each population was stained with respective antibodies indicated in Supplementary Table S 6A for myeloid lineage and HSC. Gating strategy of HSPC subsets of CMP, EMP and MEP with NS1 plot was described in Supplementary section (Supplementary Figure S2). The data revealed a fold decrease of HSC on D7 PI in comparison to D5 and D10 in DENV-infected UCB cells (Figure 1C). When compared to respective lineage progenitors (HSPC), HSC was markedly high, and other myeloid lineages were inhibited significantly (unpaired t-test was applied to compare each population, *P* < .001∗∗∗). We found a significant correlation of HSC with average viral load (*P* = .046∗). The overall population of HSCs was increased in UCB cells after DENV infection, but most of the progenitors of myeloid/megakaryocytic progenitors, such as CMPs, MEPs, were significantly decreased in total cells after DENV infection (Supplementary Figure S2.1A).

### 3.2 Functions of transcription factors GATA-1, GATA-2, and GATA-3 in DENV-infected UCBs

**Figure 2:**
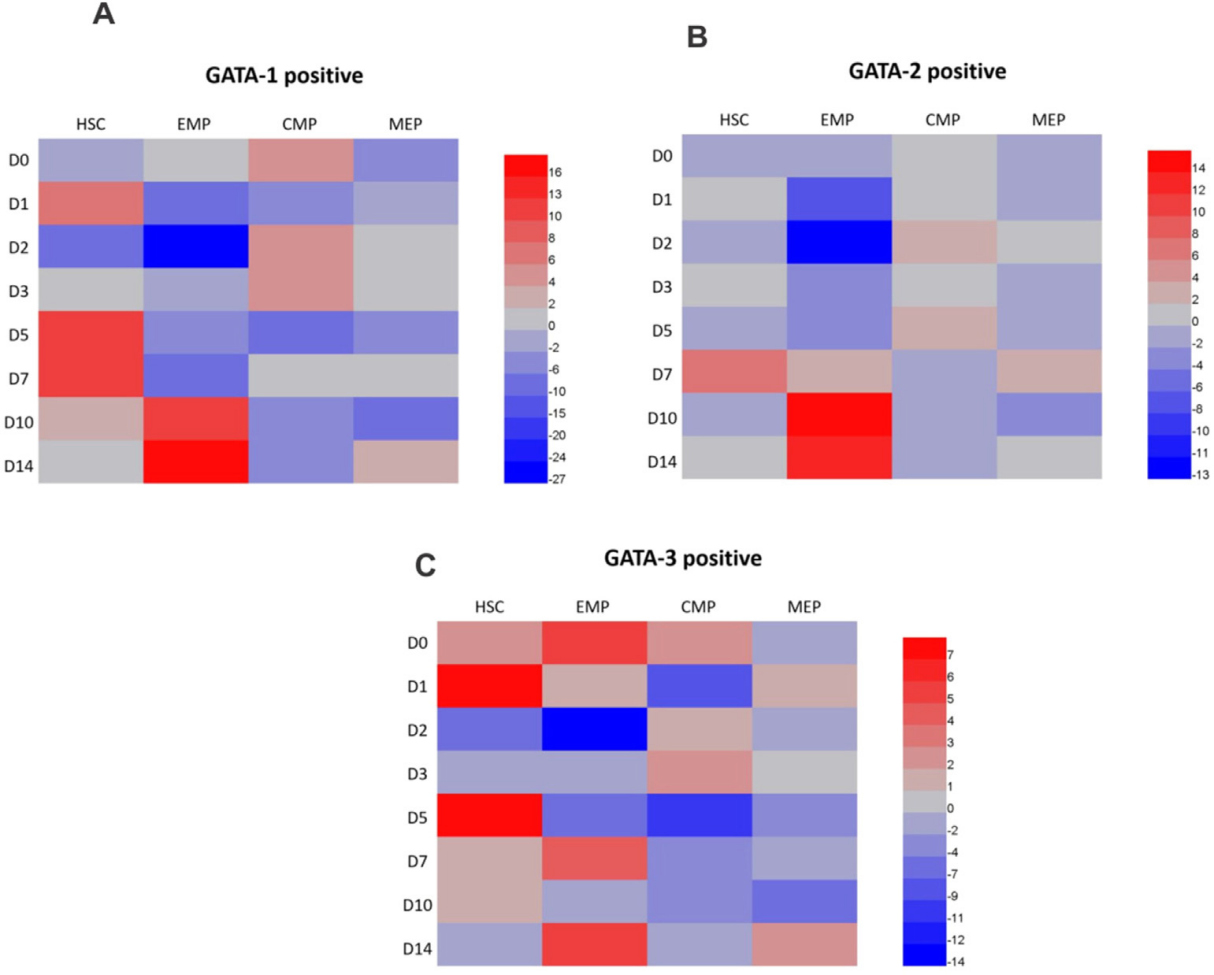
Functions of transcription factors GATA-1, GATA-2, and GATA-3 in DENV-infected UCBs. The expression of GATA-1 (A), GATA-2 (B), and GATA-3 (C) in HSCs populations of DENV-infected UCB were compared with the uninfected counterparts over time (N = 7), and the average fold changes in expression intensity were shown as a color-coded heat map. Please also refer to Supplementary Figure S2.

To explore the cause of increased expression of HSC and altered hematopoiesis in DENV-infected UCB, we investigated the potential role of GATA TFs and compared the variation in specific cell populations. To show the differential expression of lineage progenitors, we depicted the relative percentage of cell populations from each subpopulation in the form of Heat Map. Based on the low and high fold change of phenotypic expression from the normalized values, GATA-1 expression in HSC was increased in DENV-infected UCBs during early phases (D5-D7). Similarly, GATA-2 bearing HSC was also slightly increased on D7 (Figure 2 A and B), corresponding with the height of virus production at D5 and D7 (Figure 1A). In contrast, GATA-1 and GATA-2 bearing megakaryocytic lineages EMP and MEP were decreased in comparison to HSC, indicating reduced differentiation of HSC to MK. GATA -1 and GATA- 2 expressing EMP lineages were strictly inhibited in early phases from D0-D7 and became predominantly high from D10. GATA-1 bearing MEP was suppressed through the time course (D0-D10), while a slight increase of GATA-2 expressing MEP was seen on D7. Similarly, GATA-3 expressing in HSCs found to increase over time (D0-D1 and D5-D10), a high degree of GATA-3 was expressed in EMP and preventing downstream differentiation into definite lineage (Figure 2C). The three TFs also showed some proneness towards CMP during initial phases of infection, indicating an upward trend of monocytes while this lineage combated the virus, which later subsided after D3. Overall, our results suggested that the population of HSC that expressed GATA TFs increased after DENV infection, while there was an inhibition of EMP along with other progenitor cells, MEP and CMP expressing TFs in total cells of UCB (Supplementary Figure S2.1 B-D). Average values of actual number of gated cells expressing GATA-1, GATA-2 and GATA-3 obtained after gating only live cells were presented in Supplementary Table S2. It became increasingly apparent that DENV promoted HSC proliferation while inhibiting the cell population within myeloid lineage. Thus, we next investigated the capacity of the infected UCBs to differentiate into specific lineages by performing a colony-forming assay. There was no visible trend between uninfected and challenged groups. A decreasing trend in myeloid lineages CFU-GM and CFU-GEMM after DENV infection was observed, though the difference was not statistically significant (Supplementary Figure S2.2). However, the results from colony-forming assay suggested that the differentiation of myeloid progenitor cells was obviously affected during DENV infection. The detail descriptions of the protocol for colony-forming assay were provided in Supplementary Data.

### 3.3 DENV infection upregulated the expression of CD34^+^ and CD133^+^ HSCs

**Figure 3:**
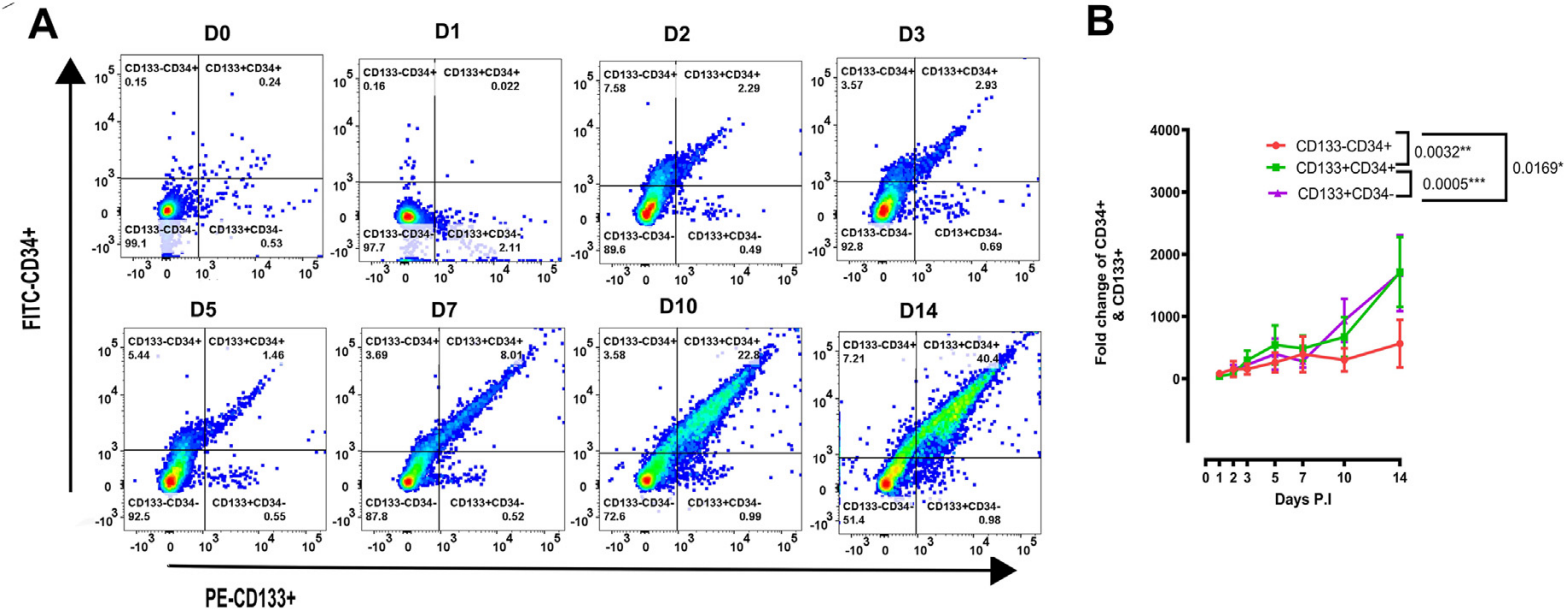
DENV infection upregulated the expression of CD34^+^ and CD133^+^ HSCs. (A) Cells were gated based on the singlet gating strategy excluding the debris for cell enumeration of CD34 and CD133 expression in each compartment. The percentage of cells in each quadrant was indicated. (B) Kinetic of the fold change expression of CD34^−^CD133^+^, CD34^+^CD133^+^, and CD34^+^CD133^-^ sub-populations in DENV-infected UCBs (N = 7). Data were normalized by their corresponding D0 (D0: 2 h post infection) from each donor to compensate for the minor differences in the initial (D0) gating percentages.

Based on the functionality of GATA TFs on distinct sub-populations, the expression of transcriptional GATA factors was found to be coordinated with the changes in HSPCs of infected UCB cells, resulting in an overall increment in HSC population. We then focused on the CD133^+^ and/or CD34^+^ HSCs surface markers in DENV-infected UCBs by MFCM. The strategy for quadrant gating of CD133^+^ and/or CD34^+^ cells is shown in (Figure 3A). Results indicated that there was a gradual increase in CD133^+^CD34^+^ subset as well as single positive CD133^+^ and CD34^+^ populations. Furthermore, a significant positive correlation was found between the absolute number of CD34^+^ and CD133^+^ cells (Figure 3B). Actual number of cells is provided in Supplementary Table S3 A-C.

### 3.4 Coordinated changes of CD133^+/-^ CD34^+/−^NS1^+^ cells in DENV-infected cord blood cells

**Figure 4:**
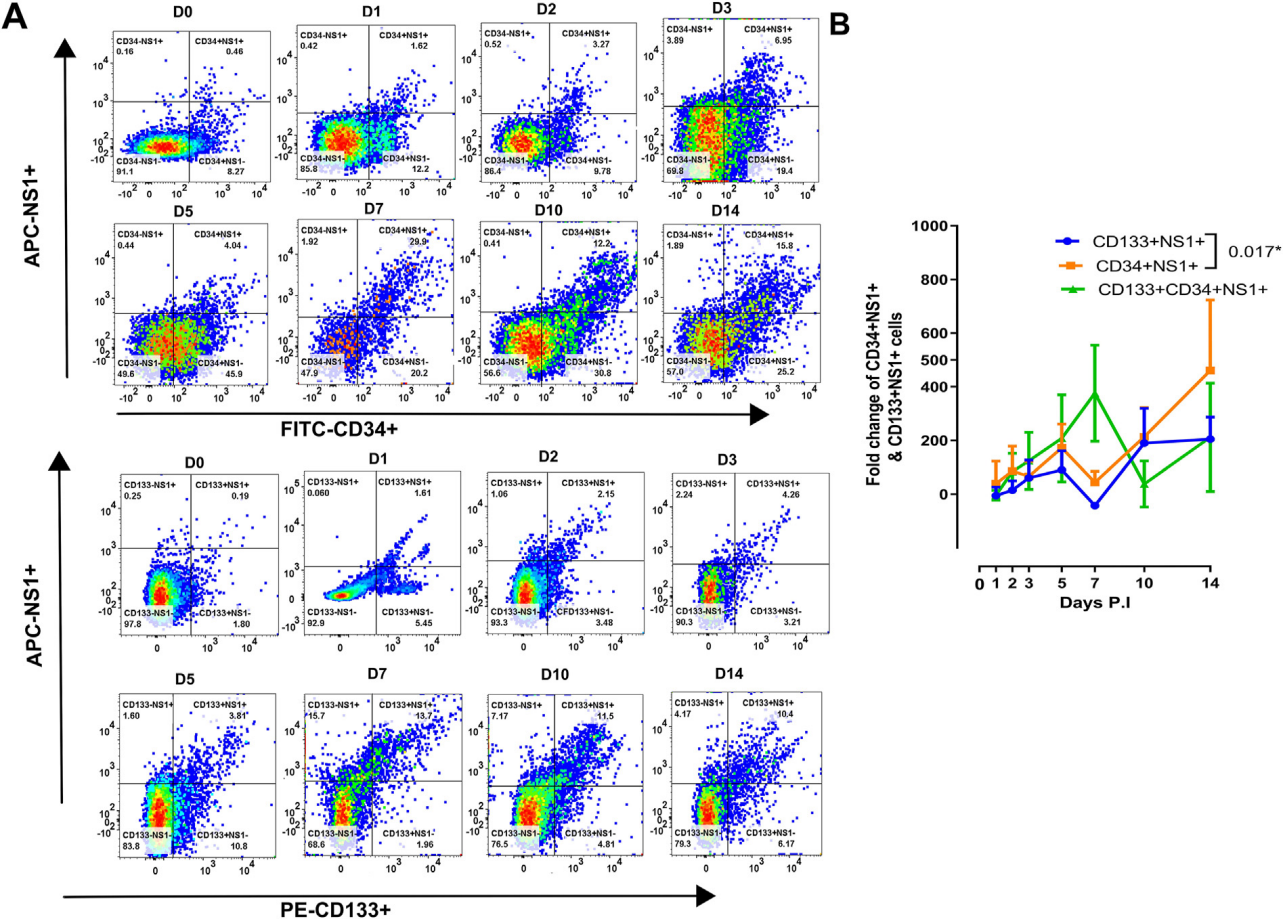
Coordinated changes of CD133^+/-^ CD34^+/−^NS1^+^ cells in DENV-infected cord blood cells. (A) Representative gating strategy of NS1^+^CD133^+^ CD34^-^ and NS1^+^ CD34^+^CD133^-^ from one donor. (B) Fold change expression of CD133^+^, CD34^+^ with NS1^+^ based on flow cytometry analysis of characteristic markers NS1^+^CD133^+^, NS1^+^CD34^+^, CD133^+^CD34^+^NS1^+,^ and the results were expressed based on gating percentage of populations. The value was interpreted by normalizing the gating percentage of each day with its corresponding D0 to recompense for the minor differences and excessive noise of NS1 signal in flow cytometry data collected afterwards (D0: 2 h post infection). Please also refer to Supplementary Figure S3.

Next, we extended our analysis to evaluate the time-related variation in subsets levels of CD133^+/-^ CD34^+/−^NS1^+^ cells in DENV-infected UCB at a different stage of infection to show the kinetic of cell content (Figure 4A). The fold changes in the gating percentage of CD34^+^, NS1^+^, and CD133^+^ demonstrated that CD34^+^NS1^+^ and CD133^+^NS1^+^ increased on D5 and decreased on D7, followed by an upward surge on D10 PI. In contrast, CD133^+^CD34^+^NS1^+^ cells had an intermittent increase on D7, a major drop on D10, and another increase on D14 PI (Figure 4B). Actual number of cells CD133 + NS+, CD34 + NS1+ and CD133 + CD34 + NS1+ cell population from DENV infected UCB obtained after gating the cells was provided in Supplementary Table S4 A-C. Representative example of flow analysis of CD133 and CD34 with NS1 expression was presented in Figure 4A and Supplementary Figure S3.1. To show the kinetic and modulation of NS1^+^ on CD133^+^ CD34^+^ double-positive cells in DENV- infected UCB, gated frequencies of NS1^+^ on CD133^+^CD34^+^ cells from one donor was presented in Supplementary Figure S3.2A. Frequencies of gated NS1^+^ were calculated as described in Supplementary Figure S3.2B. Above all, the results showed that NS1^+^ could have an impact on CD133^+^ and/or CD34^+^ populations; even though the number of CD34^+^CD133^+^ was relatively increased, indicating that NS1 may modulate the function of stem cells.

### 3.5 Characterization of NS1 with CD133^+^ and CD34^+^ cells in DENV-infected UCBs using immunofluorescence assay

**Figure 5:**
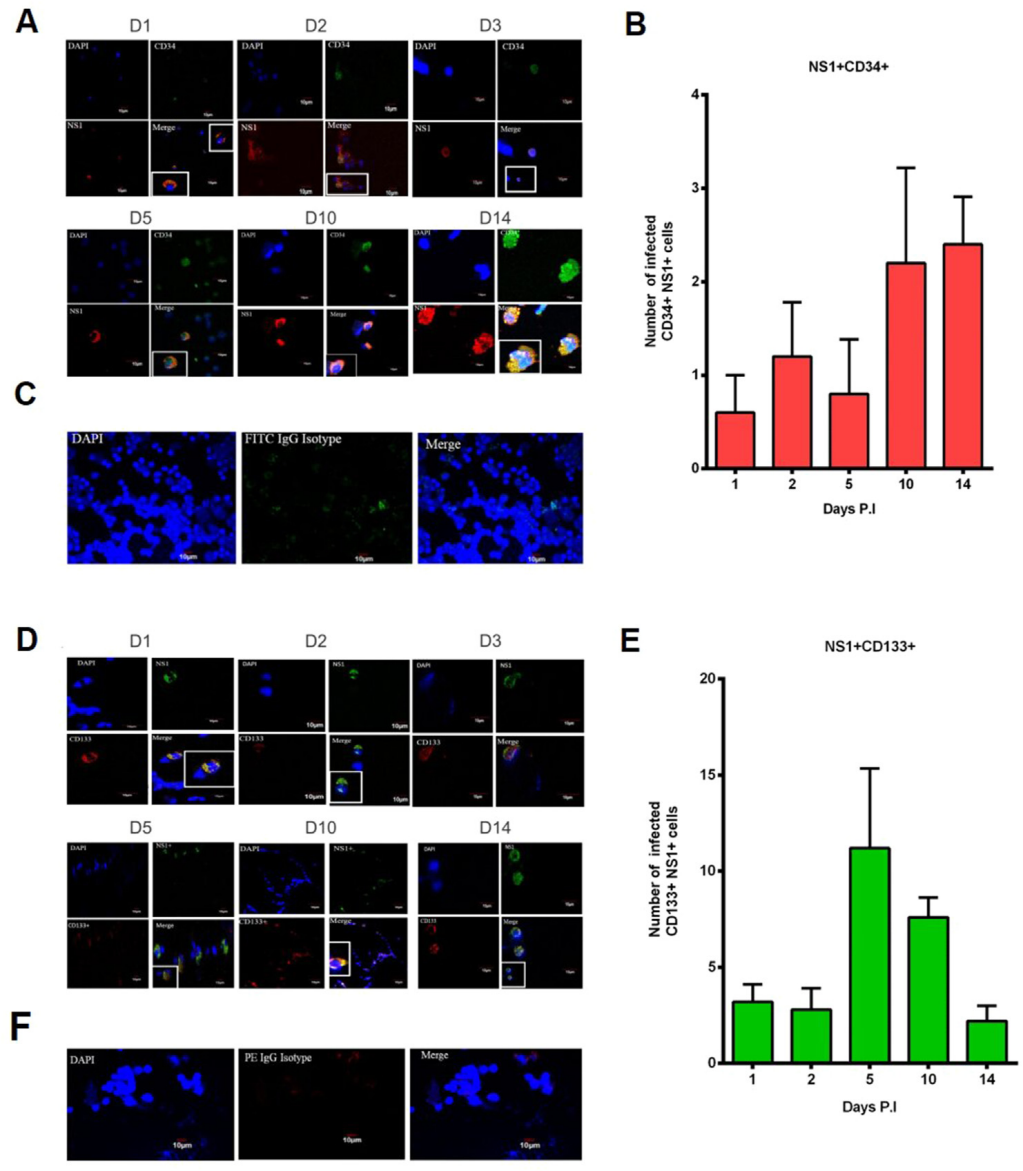
Characterization of NS1 with CD133^+^ and CD34^+^ cells in DENV-infected UCBs using immunofluorescence assay. (A) Representative fluorescent images of CD34 (FITC) and NS1 (AF 568) showing infected stem cells with associated NS1^+^. (B) Bar diagram indicating actual numbers of CD34^+^NS1^+^ cells at indicated time points. (C) FITC isotype control. (D) Sequential confocal images of CD133 (PE) and NS1 (AF488). (E) Bar diagram indicating actual numbers of CD133^+^NS1^+^ cells at indicated time points. Scale bar (=10 μm). (F) PE isotype control. Images were captured and counted in 5 fields. Original magnification was 60X in oil immersion.

**Figure 6:**
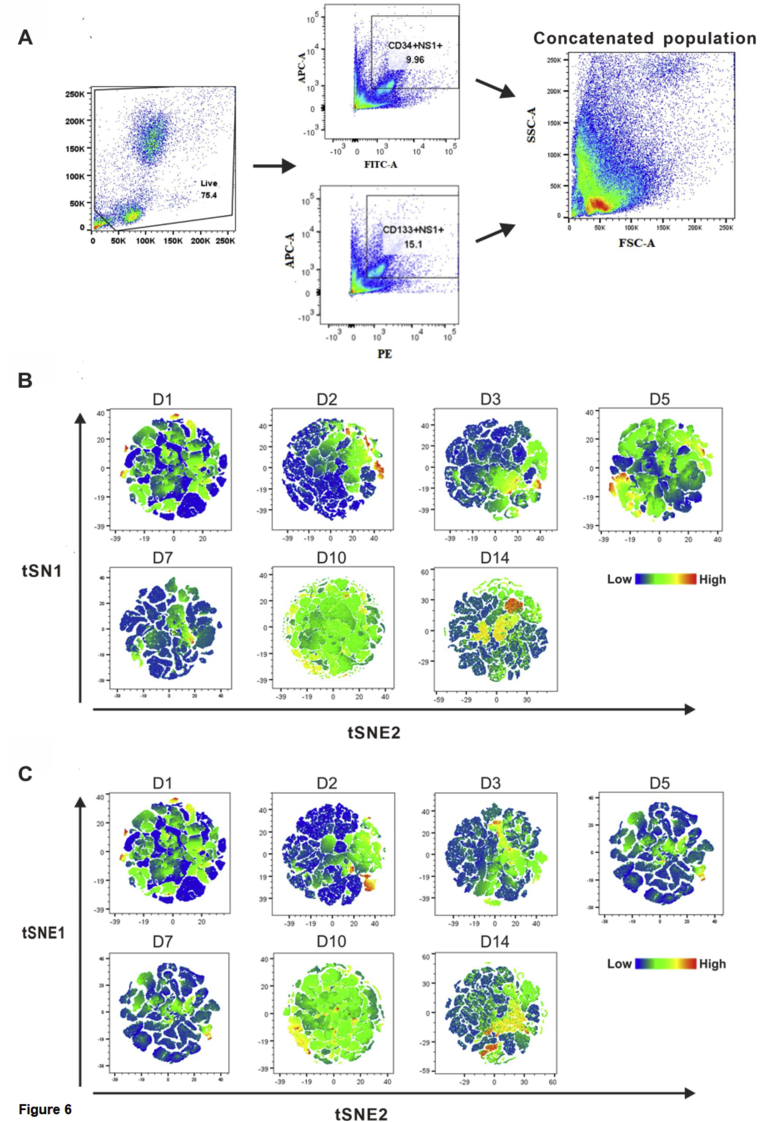
Temporal heterogeneity of CD133, CD34, and NS1 using t-SNE. (A) Representative gating strategy of CD133^+^NS1^+^ and CD34^+^ NS1^+^ double-positive cells, respectively, and concatenation of CD133^+^ and CD34^+^ at designated time points. (B and C) Projection of specially defined CD34^+^NS1^+^ and CD133^+^NS1^+^ cells on concatenated live cells. FlowJo v10 was used to calculate t-SNE. Expression intensity was indicated by the color-code bar (t-SNE was run on down sample event = 50,000, Perplexity = 100, Iteration = 1000). Please also refer to Supplementary Figure S4.

The fluorescence assay demonstrated that NS1 was expressed in CD34^+^ cells which reached the maximum after D10 PI (Figure 5 A and B). In addition, NS1 was also expressed in CD133^+^ cells, and the peak of NS1 expression was on D5 PI (Figure 5 D and E). Mock controls for CD34^+^ and CD133^+^ cells were performed in parallel (Figure 5 C and F). As expected, CD34^+^NS1^+^ IFA data coincided with the results observed in MFCM analysis. On the contrary, there was a subtle discrepancy in the data of NS1^+^CD133^+^ on D10. This form of CD133 antibody was probably not properly detected in all the fields by the fluorescent secondary antibody on D10 in immunostaining, and, therefore, the total number of infected cells per field was less comparable to D5 and diverged from MFCM data (Figure 4B). We rationalized that due to excessive proliferation of CD133 on D10 as shown by the following t-SNE analysis (Figure 6C); DENV might have affected the binding of antibodies to infected stem cells presumably by reducing the binding affinity to surface proteins.

### 3.6 Temporal heterogeneity of CD133, CD34, and NS1 using t-SNE

To better understand the variation in expression of CD34^+^ or CD133^+^ cells with NS1, we used a dimensionality reduction technique t-SNE (t-Distributed Stochastic Neighbor Embedding) on a dataset (N = 7) to visualize the distribution of CD133 and CD34, as well as their co-expression with NS1 (Figure 6A). This technique enabled us to create a t-SNE map the distribution levels of CD34^+^ NS1^+^ and CD133^+^ NS1^+^ among the different time points during DENV infection in UCBs (Figure 6 B and C) (The peak intensities of CD34^+^ NS1^+^ and CD133^+^ NS1^+^ were higher on D10 and D14, corresponding to the fold increased gating kinetics from MFCM (Figure 4B). Notably, the expressions of CD34^+^ NS1^+^ and CD133^+^ NS1^+^ cells were decreased on D7. The decrease of double-positive subsets could be a consequence of increasing triple-positive CD133^+^CD34^+^NS1^+^ population (Supplementary Figure S4). The t-SNE maps demonstrated that CD133^+^CD34^+^NS1^+^ cells were susceptible to becoming triple-positive on D7 when the viral titer was high but then decreased at D10 PI (Supplementary Figure S4 A and B). Further, to explore the association of NS1 with different lineages of stem cells, mapping of functionally defined groups of sub-populations confirmed elevated expression of HSC (Supplementary Figure S4.1). Differences in levels of EMP, CMP, and MEP were also evident with notably higher HSC expression.

### 3.7 Confirmation of the infectivity of virus in specific sorted CD133^+^ and CD34^+^ cells as well as the recovery of infectious virus from these cells

**Figure 7:**
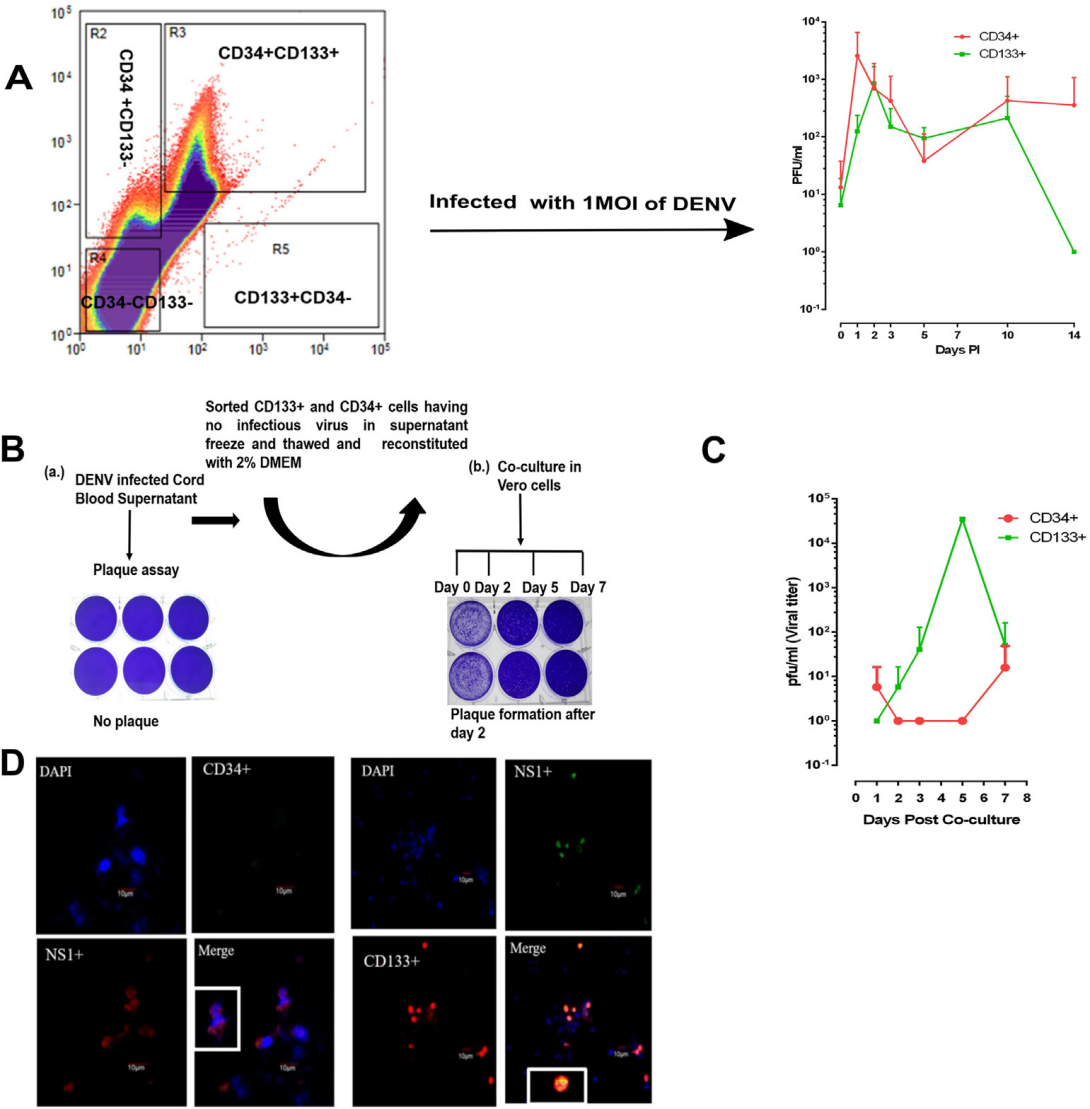
Confirmation of the infectivity of virus in specific sorted CD133^+^ and CD34^+^ cells as well as the recovery of infectious virus from these cells (A) Schematic representation of sorting and enrichment of CD133^+^ and CD34^+^ stem cells and viral load from the supernatant (n = 6). (B) Schematic presentation of the co-culture system. Infected CD133^+^ and CD34^+^ cells obtained from each time point with no infectious virus in the supernatant were co-cultured with Vero cells. (C) The replication curve of the recovered virus from infected CD133^+^ and CD34^+^ cells (n = 5). (D) Immunofluorescence staining of CD133^+^ and CD34^+^ with NS1^+^ obtained from co-culture.

To verify the infectivity of DENV in CD133^+^ and CD34^+^ cells, the cells were respectively sorted out from UCBs. More than 1 × 10^6^ cells were obtained after sorting from 1×10^8^ total UCB. They were subjected to DENV infection, and the supernatants were collected for plaque assay at indicated time points. The average loads of infectious virus in supernatants of sorted CD34^+^ or CD133^+^ cells are shown in Figure 7A. The viral titer in sorted CD34^+^ cells from different UCB donors varied as clearly shown in Table 1. The sorted CD34^+^ cells produced ample virus up to D14 with 8500 pfu/ml at D1 in donor 1, whereas in donors 2, 4 and 5, there was no detectable virus in the supernatants of sorted CD34^+^ cells. In contrast, viral loads in supernatants of sorted CD133^+^ cells were more notable in most of the donors except for donor 2 (Table 1). Supernatants obtained from sorted CD133^+^ cells from donor 1 were contaminated after D2; hence the plaque assay was not performed. We also amplified the recovery rate of progeny virus from DENV-infected CD133^+^ cells and CD34^+^ cells to effectively obtain infectious virus in the supernatants. Based on the recorded values of viral titers shown in Table 1, DENV-infected CD34^+^ or CD133^+^ cells from 5 donors (Donor 2-Donor 6) were subjected to co-culture with Vero cells (Figure 7B). The titer of virus recovered through co-culture is shown in Table 2. We observed that Donor 3 had a detectable yield of the virus in the supernatants of infected CD133^+^ cells at D5 with 100 pfu/ml, but no detectable viral outputs in the supernatants of infected CD133^+^ cells were observed from D7 to D14 (Table 1). Unexpectedly, the same CD133^+^ cells from D7 were able to produce a large quantity of virus with a titer of 1.7 × 10^5^ pfu/ml after 5 days of co-culture (Table 2). Similarly, donor 3 had no virus yield from D10 in supernatants of DENV-infected CD34^+^ cells. Upon co-culturing the CD34^+^ cells, the supernatants contained a marginal production of virus with about 100 pfu/ml up to day 7 (Table 1, Table 2). These results indicated that the CD133^+^ and CD34^+^ cells were not only infectable by DENV but could also serve as the reservoir for the virus (Figure 7C). The infectivity of DENV in the sorted CD34^+^ and CD133^+^ cells were further corroborated by IFA staining, in which both cells were shown to express NS1 protein. IFA staining also showed that CD133^+^ cells had a higher number of NS1 co-expressing cells as compared to CD34^+^ cells (Figure 7D). The virus recovery from CD133^+^ cells was more notable in co-culture with Vero cells at day 5 and sustained up to day 7 as compared to CD34^+^, which increased on day 7 in donor 3. This reveals that CD133^+^ cells are more spontaneous in virus reactivation and more susceptible to active infection, whereas CD34^+^ cells are not very significant in reactivation and predominantly latent.

**Table 1:** Viral titers from sorted CD133^+^ and CD34^+^ cells after DENV infection.

	Donor 1		Donor 2		Donor 3		Donor 4		Donor 5		Donor 6	
Days P.I.	CD34^+^	CD133^+^	CD34^+^	CD133^+^	CD34^+^	CD133^+^	CD3^+^	CD133^+^	CD3^+^	CD133^+^	CD34^+^	CD133^+^
0	50	0	0	0	100	0	0	0	0	0	0	0
1	8500	1	0	0	250	250	0	25	0	75	1512.5	0
2	250	100	0	0	2500	350	0	175	0	1800	25	13
3	213	∗	0	0	1475	150	0	1215	0	38	0	38
5	0		0	0	150	100	0	375	0	163	0	63
7	0		0	0	275	0	0	50	0	625	0	50
10	0		0	0	0	0	0	0	0	0	0	225
14	1425		0	0	0	0	0	0	0	0	0	0

∗Supernatants obtained from sorted CD133+ cells from donor 1 were contaminated after Day 2.

**Table 2:** Viral load after co-culture.

	Donor 2		Donor 3		Donor 4		Donor 5		Donor 6	
	CD34^+^CD133^+^		CD34^+^ CD133^+^		CD34^+^CD133^+^		CD34^+^CD133^+^		CD34^+^CD133^+^	
Co-c. d^a^	14		10	7	10		14		14	
**D.P. Co-c**^b^										
2	0	0	12.5	200	12.5	0	0	0	0	25
5	0	0	25	175000	0	0	0	0	0	0
7	0	0	100	250	0	0	0	0	0	0

a Co-c. d: Day of Co-culture.

b D.P Co-c: Days Post Co-culture.

## 4 Discussion

In this study, we demonstrated that human UCBs were highly permissive to DENV infection. Studies have described the presence of preinfectional antibodies and phenomena of enhancement of DENV antibodies in cord blood [47, 48]. Our observations, although from a small sample size, revealed that HUCB serum from each participant was serologically negative for DENV IgG and IgM, which coincides with a previous report suggesting that the prevalence of DENV antibodies in the general population in Taiwan is low (Supplementary Table S5) [49]. Indication of IgM and IgG antibodies in cord blood serum of neonates is suggestive of transplacental transfer of DENV and transfer of maternal IgG antibodies [50]. In recent years, it has been explored that hematopoietic cell candidate expresses FcRn receptor to recycle internalized IgG and maintain IgG level [51, 52]. Studies of infants with dengue in which cord blood serum is used to define the entity of the disease enhancement [47] will, therefore, not discern the relative infectivity of invading hematopoietic cells as well as antibodies-producing cells from the mother. To better comprehend cell-associated virus infectivity, we emphasized the potential importance of stem cells in UCB to demonstrate a possible route of perinatal transmission. Previously published reports have shown the impact of congenital infection on the markers of hematopoietic progenitor cell potency [53]. Besides, the association of DENV antigen with trophoblast, stroma, and decidua in placental tissue has been reported [54]. However, no reports are suggesting that HSCs in HUCB are also capable of getting infected by DENV, which could also play a role in vertical transmission.

In line with this notion, we hypothesized that hematopoietic stem cells in UCB are the first potential target to be exploited by the virus, leading to congenital infection. We evaluated the myeloid lineage as the target for DENV in UCB cells. Our results revealed that HSCs increased after DENV infection in *in vitro* analysis of UCB.

In earlier reports, viral hematodepressive disease without serological identity has been diagnosed in Thai children [55]. Infants with primary dengue had significantly lower platelet nadirs and greater hemoconcentration [20]. This raises the distinct possibility that certain aspects of hematopoietic response to virus infection remain obscure. As reported, GATAs are the transcription factors that operate sequentially in the activation and repression of genes in the development of blood cells and orchestrate cell differentiation and proliferation [56, 57]. The phenotypic and functional characteristics of GATA TFs investigated in our study suggested that hematopoietic GATA factors could be intertwined with the differentiation of HSC in DENV-infected UCB, as documented in prior study [58]. DENV-associated thrombocytopenia caused the inhibition of HSC differentiation to MK development in BM to reduce the production of platelets. Combining our results, we found that there was an altered expression of these TFs that changed the balance of transcription factors, which may explain the disordered hematopoiesis due to DENV infection (SF2). This implies that in early infancy, the maturation arrest of HSC caused by DENV infection in UCB leads to inhibition of the myeloid lineage and predisposes HSCs to interact with DENV, thereby potentially causing damage to MK production. The fact that protection provided by maternal dengue antibodies lasts 6 months or less in infants, consequently leaving them susceptible to dengue virus infection [47], and causing increased vascular permeability compared to adults, raises the question of whether other sensitized cells with specific virus receptors may mediate infection from mother to the fetus.

There is growing evidence that hyaluronan receptor CD44 plays a substantial role in the increased vascular permeability and decreased platelet count associated with severe dengue infection [59, 60]. Considering the evidence, that CD44 as a provocative mediator in severe dengue, we sought to investigate the role of CD44 on DENV-infected UCB. In this study, CD44 was highly expressed in cord blood, because of aberrant expression of CD44, could be a nexus to the DENV production in stem cells, affecting the proliferation and differentiation activity and may implicate in involving maintenance of stem cell proliferation and differentiation activity (Supplementary Figure S5 A-D).

It is thought provoking that there is a perturbation of GATA1-3 at D3, opposite to the increase of CD44^+^CD133^+^ observed on D3. Consequently, on D5 and D7, when the GATA1-3 levels increased, while CD44^+^CD133^+^ expression simultaneously decreased, and the viral peak titer was high. This result indicates that CD44 regulation may occur at the transcriptional level contributing to the early infection event However, how GATA-3 regulates HSC remains to be determined According to literature, GATA-3 is dispensable for HSC regulation and generation of early T cells [61, 62]. It has been suggested that CD44 acts as an early marker of differentiation on immature cells CD34^+^ and supports HSC survival and maintenance of proliferating hematopoietic progenitor cells [63, 64, 65]. We observed a longitudinal increase in the CD44 rate among CD133 and CD34 cells from days 2–14 (Supplementary Figure S5D). We could conjecture that CD44 might be sequestered and suppressed in stem cells at D5 and D7 compared to non-stem cells, which was either the result or the cause of the innocuous DENV infection. Through analysis of DENV infected HSCs using MFCM, the highest fold increase of CD133^+^NS1^+^ and CD34^+^NS1^+^ was reached around D14. This possible mechanism for the restraint in differentiation that leads to an increase in the kinetic of CD133^+^ and CD34^+^. However, correlating protein levels of various isoforms of CD44 and stem cell-related genes with NS1 will require to be further confirmed.

There was an inverse kinetic trend of CD133^+^CD34^+^NS1^+^ as compared to CD34^+^NS1^+^ and CD133^+^NS1^+^ (Figure 4B). We reasoned that DENV provided a survival signal to promote double positive CD133^+^NS1^+^ and CD34^+^NS1^+^ cells to display triple-positive phenotype when the viral titer was high on D7, by increasing their proliferation and preserving their repopulating ability. However, the exact mechanism behind this remains to be explored. We also attempted to observe the space and temporal changes in NS1^+^ with CD133^+^ and CD34^+^, respectively, using t-SNE. NS1^+^CD133^+^ and NS1^+^CD34^+^ expressions were initially low but later changed and gradually reached a maximum on D10, followed by contraction on D14 after infection. There is evidence of HCV infecting HSC and measles virus suppressing HSC through direct infection of CD34 [66,67]. Based on these assumptions, CD133^+^ and CD34^+^ sorted cell was infected with DENV Prevalence of possible HCV virus contaminants in UCB units has been reported to be a source of virus transmission risk from mother to the fetus and establishment cytomegalovirus latency in HPCs [68, 69]. In accordance with this interpretation, we observed that DENV was detectable in CD133^+^ and CD34^+^ sorted cell populations and produced a high viral load in certain donors, while some maintained low viral loads leading to viral persistence (Table 1). Considering this fact, many cases of congenital dengue infection have occurred in neonates born to mothers infected very late in pregnancy, with the latent period of dengue reported to be 3–15 days [31]. We submitted that the virus might be present in cells in a latent state and potentially reactivated if cellular conditions changed; hence, we co-incubated infected UCBs with Vero cells, which are infectable by DENV. CD133^+^ cells had a higher propensity to recover the virus than CD34^+^ cells did, suggesting a potential phenotype for latent infection. We recovered virus from CD133^+^ as well as from CD34^+^ cells, suggesting the establishment of latent infection in these specific cells, which could potentially confer vertical transmission and reactivation of the virus.

Lastly and most importantly, one of the donors in our study whose serological test scored negative anti-DENV IgG and IgM had dengue fever and delivered twins (Mother 8). One infant was normal and the other had dengue infection. In the plaque assay using the infectious culture supernatant of cord blood, twin A with DENV infection produced a high viral load in sorted CD34^+^ and CD133^+^ cells, in contrast to twin B with no virus production. From this result, we speculated that stem cells in twin B could have other unknown factors contributing to the insusceptibility of CD34^+^and CD133^+^ to DENV infection. Literature suggest that there is an individual variation to DENV infection among twins, which is consistent with the previous report [70]. The influence of maternal age on UCB HSC concentration as well, reinforces the speculation for UCB challenging to be infected [71]. However, our in vitro study cannot fully attribute a substantial role to HSCs in maternal-fetal transmission. Our approach needs to be further explained by animal modeling or in vivo studies.

## Declarations

### Author contribution statement

Guey Chuen Perng: Conceived and designed the experiments; Wrote the paper.

Amrita Vats: Conceived and designed the experiments; Performed the experiments; Analyzed and interpreted the data; Wrote the paper.

Tzu-Chuan Ho, Irwin Puc, Chiung-Hsin Chang: Contributed reagents, materials, analysis tools or data; Yi-Ju Chen: Performed the experiments.

Yu-Wen Chien: Analyzed and interpreted the data.

### Funding statement

This work was supported by Taiwan 10.13039/501100004663Ministry of Science and Technology (MOST-106-2321-B-006-012; MOST 107-2314-B-006-MY3 and MOST 103-2320-B-006-030-MY3).

### Data availability statement

Data included in article/supplementary material/referenced in article.

### Declaration of interests statement

The authors declare no conflict of interest.

### Additional information

No additional information is available for this paper.

## Appendix A Supplementary data

Vats_et at_Supplemmentary_Revised_R2 Vats_et at_Supplemmentary_Revised_R2
