# Supplementary material for: Evidence that hematopoietic stem cells in human umbilical cord blood is infectable by dengue virus: proposing a vertical transmission candidate
Source: Heliyon. 2021 Apr 20;7(4):e06785. doi: 10.1016/j.heliyon.2021.e06785 (PMC8082560; doi:10.1016/j.heliyon.2021.e06785)
Supplement: Vats_et at_Supplemmentary_Revised_R2 [file mmc1.docx]

**Supplementary Data**

**Detailed description of the protocol used in the study.**

***Human UCB are infectable by dengue virus****.*

1*10^7 UCB cells from each donor (N=7) were subjected to dengue viral infection. We collected the supernatant of DENV-infected UCB (1 MOI) at the respective time points (D0-D14) and subjected to standard plaque assay. A 10-fold dilution of infectious supernatant was prepared from different time points, and 0.4 ml aliquots were inoculated into susceptible monolayers of BHK cells in the 6-well plate. After the incubation period of 2 hrs, while shaking for every 15 min to allow the virus to attach to cells, the monolayer was covered with 2% MC medium (Methyl cellulose without antibiotics). The plates were harvested after 7 days of incubation to observe the release of progeny virus from DENV-infected cord blood cells. The titer of the infectious virus was calculated in PFU/ml. Viral replication kinetics of DENV-infected UCB indicated that UCB could be efficiently infected by DENV and support the infection **(Figure 1A and Supplementary Figure S1),** though a variation in the individual donors was observed (**Supplementary Table S1)**

***Immunophenotyping and Multicolor flow cytometry analysis (MFCM)***

The cells harvested at indicated time points were subjected to MFCM by staining with fluorescence-conjugated antibodies for phenotypic analysis of hematopoietic stem and progenitor cells (HSPC) in infected and uninfected cells. All staining was performed in PBS containing 1% BSA mouse anti-human surface marker antibodies, specific for the HSC (CD34, CD133) and sub-populations of myeloid cell lineage (CD45RA, CD61, CD41a, and CD14) with their respective isotype controls **(**obtained from BD Biosciences**).** After staining for 30 min with surface marker antibodies on ice, cells were washed with PBS containing 0.1% BSA, permeabilized with 0.1% saponin followed with NS1 (AF-647, BD Biosciences) intracellular staining for 1 h. The excitation and emission maxima of AF-647, are identical to APC. We tended to acquire the NS-1-stained cells after intracellular application using APC- channel in BD-LSR Fortessa. For the analysis of GATA TFs (**Figure 2A-C**), 1x10^7^ cells from both the infected and uninfected cells were co-stained with antibodies for cell surface markers as well as intracellular staining of GATA1/2/3 (Research and Design**,** panel). Subsequently, all stained cells were washed in PBS containing 0.1% BSA and kept in dark at 4° until they were analyzed. The cells were acquired using LSR Fortessa (BD Bioscience) for MFCM analysis, and the gated cells were analyzed by using Kaluza software and Flow-Jo. To compare the expression of different cell types and GATA TFs, cells were divided into two groups, namely DVI (infected) and DVU (uninfected). To evaluate the magnitude of stem cells population, we normalized the value of all the subsets by subtracting the value of infected (I) gating percentage from uninfected (C) gating percentage as (I-C)/C. We presented the data here in the fold change expression of staining intensity by averaging the gating percentage of all the subsets from 7 donors and clustered together in the classified subpopulation (stem cell lineage, **Figure 1B**).

***Immunofluorescence staining and confocal image capturing****.*

To analyze and confirm the association of NS1 expression with CD133^+^ or CD34^+^ cells in DENV-infected UCBs, immunofluorescence staining (IFA) was performed from D1 to D14. The cells were kept for blocking in PBS with 1% BSA for 60 min at 37°C, followed by incubation with primary antibodies CD34-FITC and CD133-PE (BD Bioscience) in 1:2500 dilution for 1 to 2 h at 4ºC. The cells were then washed twice in PBS, then fixed with 4% paraformaldehyde, and attached to slides via cytospin (Thermo Scientific, cytospin 4) at 400 rpm for 8 min. The monolayer was washed twice in PBS for 5 min each, permeabilized (0.3% Triton X-100 with 1% BSA in PBS) for 30 min, and finally washed twice in PBS. The cells were labeled using NS-1 rabbit polyclonal antibody (Gentex, Taiwan) at a dilution of 1:2000 in permeabilization buffer at 4ºC for 2 h and washed twice again in PBS. They were further incubated with AF-488-donkey anti-rabbit **(**Invitrogen**)** and AF568-goat anti-rabbit (Life Technologies) secondary antibodies for 1 to 2 h in the dark at room temperature and covered with antifade reagent DAPI (**Figure 5**). Fluorescent images were captured in an Inverted Confocal Microscope FV-1000 (Olympus).

# *Colony- forming assay.*

To investigate the probable effects on the development of specific lineages during DENV infection of UCB. We used colony- forming assay to evaluate the effect of DENV infection on the differentiation of HSPCs (CD133 ^+^and/or CD34^+^) derived from UCB. Thirty-five hundred HSPCs were sorted out from UCB by magnetic beads (Miltienyi Biotech). Cells were exposed to DENV with a high value of MOI=10 for 2 hours, respectively. After 2 hours, the cells were seeded in semisolid medium to form colonies for 2 weeks, including uninfected cells. We sorted out 3,500 cells from UCB and resuspended them in 500µL Cell Resuspension Solution in eppendorf tubes before adding them into a 3 cm culture dish containing 3 mL Human Methylcellulose Complete Media (R&D systems). The 3 cm culture dish was further placed inside a 10cm culture dish containing sterilized deionized water (ddH2O) and was incubated at 37°C for 14 days. After 14 days, the colonies were characterized and scored according to the morphology following the manufacturer's instructions. An inverted microscope at 40x or 100x magnification with a culture dish marked with a scoring grid was used for further colony sub-classification. The colonies were classified into 6 categories, including colony-forming unit-erythroid (CFU-E), burst-forming unit-erythroid (BFU-E), colony-forming unit-granulocyte (CFU- G), colony-forming unit-macrophage (CFU-M), colony-forming unit-granulocyte, macrophage (CFU-GM), colony-forming unit-granulocyte, erythrocyte, macrophage, megakaryocyte (CFU-GEMM). A manual differential cell counter was used to enumerate the colonies. The total number of colonies formed from 3,500 cells were enumerated for different categories. The number of the colonies formed by the fixed number of input cells provided preliminary information on the ability of progenitors to differentiate and proliferate. The percentage of each category of colonies with respect to the total number of CFU was calculated (**Supplementary Figure S2.2**)**.** After scoring according to the morphology from each representative plate, we enumerated the colonies based on classified categories and found that CFU-M, CFU-GM, and CFU- GEMM were decreased after DENV infection. Uninfected groups were examined to visualize the difference in differentiation potential between infected and uninfected groups. Indeed, the results from colony-forming assay suggested that the differentiation of myeloid progenitor cells was obviously affected the most during DENV infection.

***Temporal heterogeneity of CD133, CD34, and NS1 using t-SNE****.*

Specific t-SNE maps were generated using measured markers of CD133^+^ and CD34^+^ in DENV-infected UCB (**Figure 6**). To depict the phenotypic variation of CD133^+^ and CD34^+^, we first generated down sample of CD34^+^NS1^+^ and CD133^+^NS1^+^with 50,000 cell events and optimized the number of iterations=1000 and perplexity =100 to separate the cell population. The run time was approximately 1 hr. With this input of parameter, the cell populations were condensed and grouped together. To visualize the peak intensity of surface marker expression of CD133^+^ and CD34^+^ on CD133^+^NS1^+^ and CD34^+^NS1^+^ subsets, fluorochrome-labeled CD133^+^ marker and CD34^+^ marker was selected on each double-positive population. Increasing cell densities were indicated by increasing color intensities, and the coloring contour plot was used. At day 10, cells were discretely separated into islands **(Supplementary Figure S4).**

In an effort to distinguish other distinct cells or another lineage different from stem cells, and to further explore the association of NS1 with CD45, CD61, CD41, and CD14 populations, we converged the different subsets with CD133 and CD34 of megakaryocytes, monocytes in clusters of classified sub-populations of HSC, EMP, CMP, and MEP to show the increment of HSC **(Supplementary Figure S4.1)** with recommended parameter values (Down sample =10,000 cell events, iteration=1000 and perplexity =50).Run time was 2 hrs.

***Flow cytometry of CD44 in DENV infected UCB****.*

For flow cytometry, UCB cells were infected with 1 MOI of DENV and cultured for 14 days (D0-D14) in 10% RPMI. Following harvesting of cells from each time point, cells were incubated with fluorescence-conjugated anti-CD44 (APC-H7), anti-CD133(PE), and anti-CD34 (FITC). Mouse IgG1κ isotype control APC-H7 (BD), mouse IgG 1 κ PE(BD), mouse IgG 1κ FITC (BD) were used as negative control, respectively. Flow cytometric analysis was performed using Flow Cytometer- BD FACS CANTO II (**Supplementary Figure S5-A-D**).

**Supplementary Figures**

**Supplementary Figure S1**


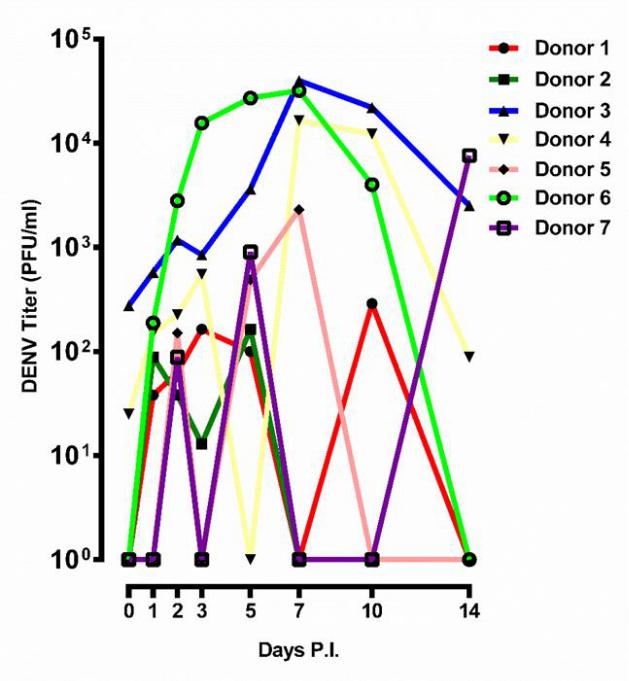


**Figure S1. Viral titer from each donor. Related to Figure 1A.** Legend as in Fig 1. Human UCB are infectable by dengue virus. 2*10^7 UCB from each donor (N=7) were performed for dengue viral infection at indicated time point. D0, 2 hrs of post infection.

**
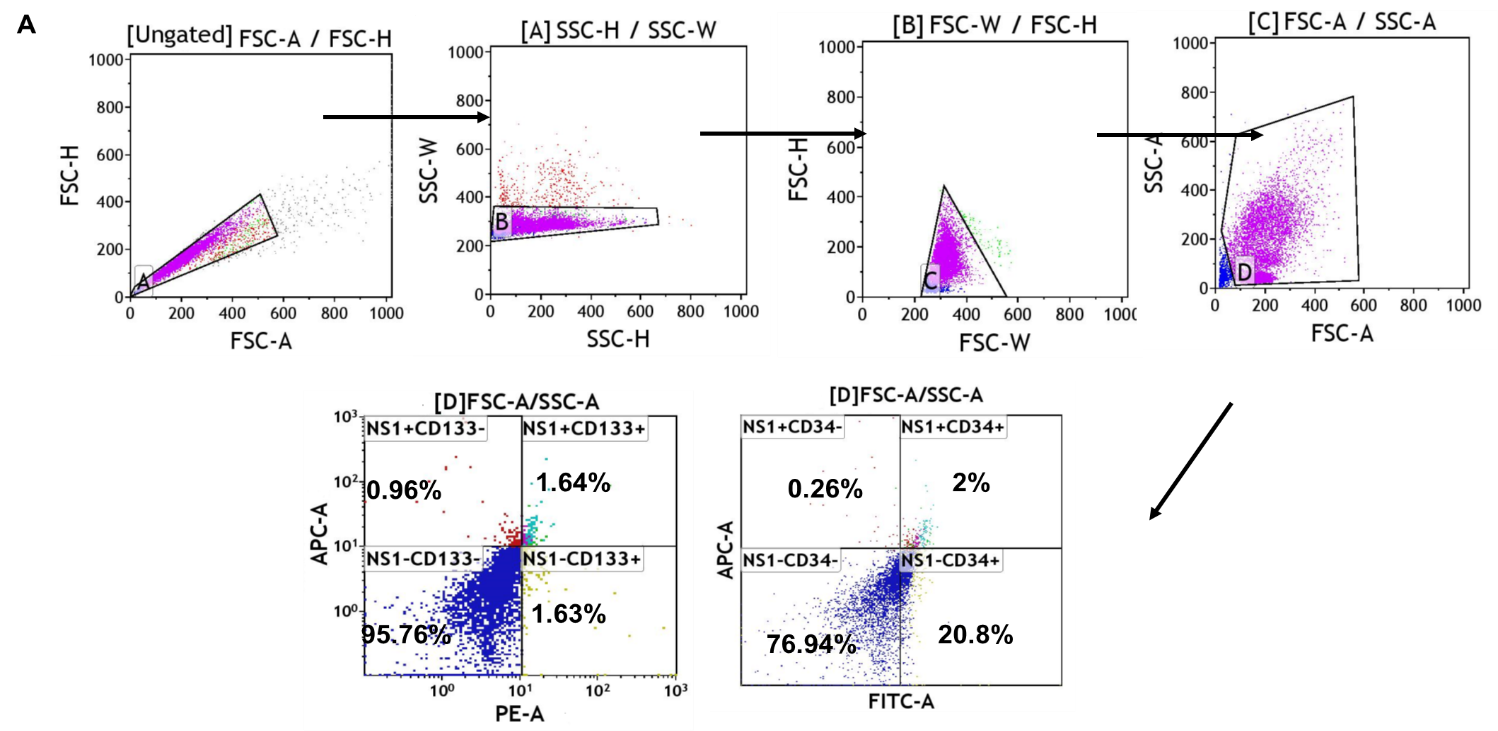
 Supplementary Figure S2**

**
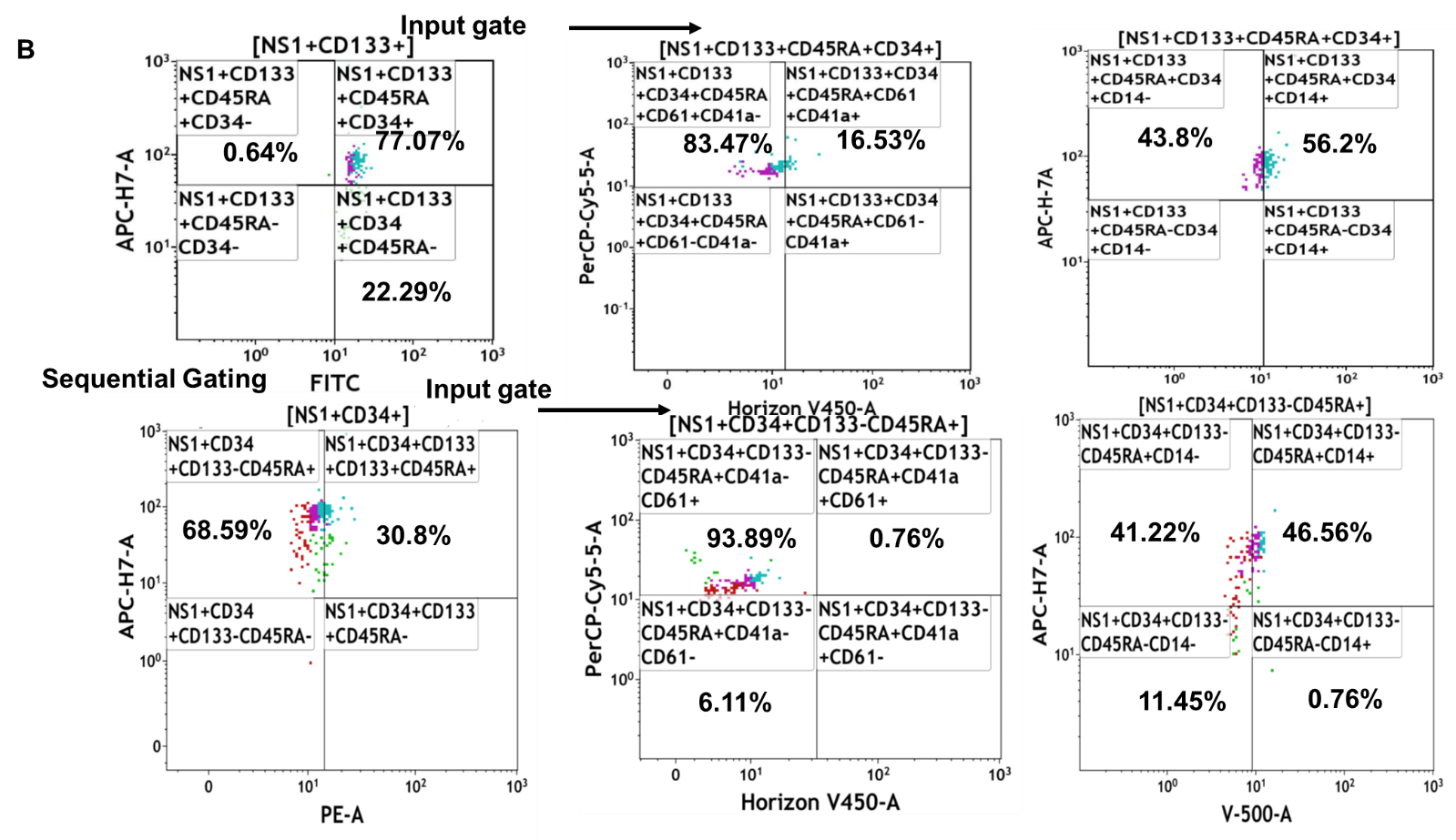
**

**
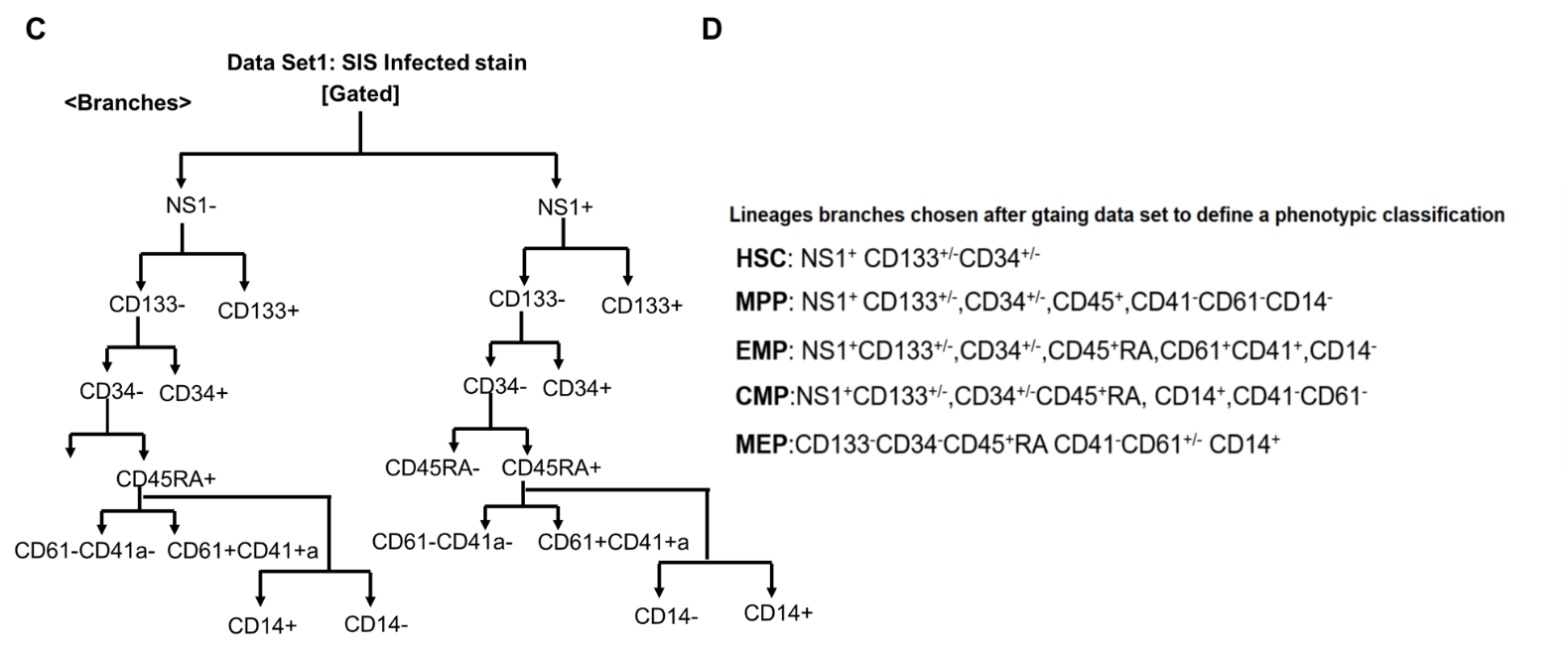
**

**Figure S2**: **Gating strategy used to isolate HSPC populations in NS1+ stained DENV infected UCB**. (A). Cells were first gated for singlets (FSC-A vs FSC-H, FSC-W vs FSC-H, SSW vs SSSH and whole cell FSC-A and SSC-A. (B). Sequential gating of each expression marker from parent gate one by one and populations are identified subjectively by positioning gates on the displayed quadrant gating**.** (C). A tree plot was represented after analyzing and gating for each phenotype using kaluza software. (D). Summary of defining cell surface markers used to separate HSPC subsets.

**Supplementary Figure S2.1**


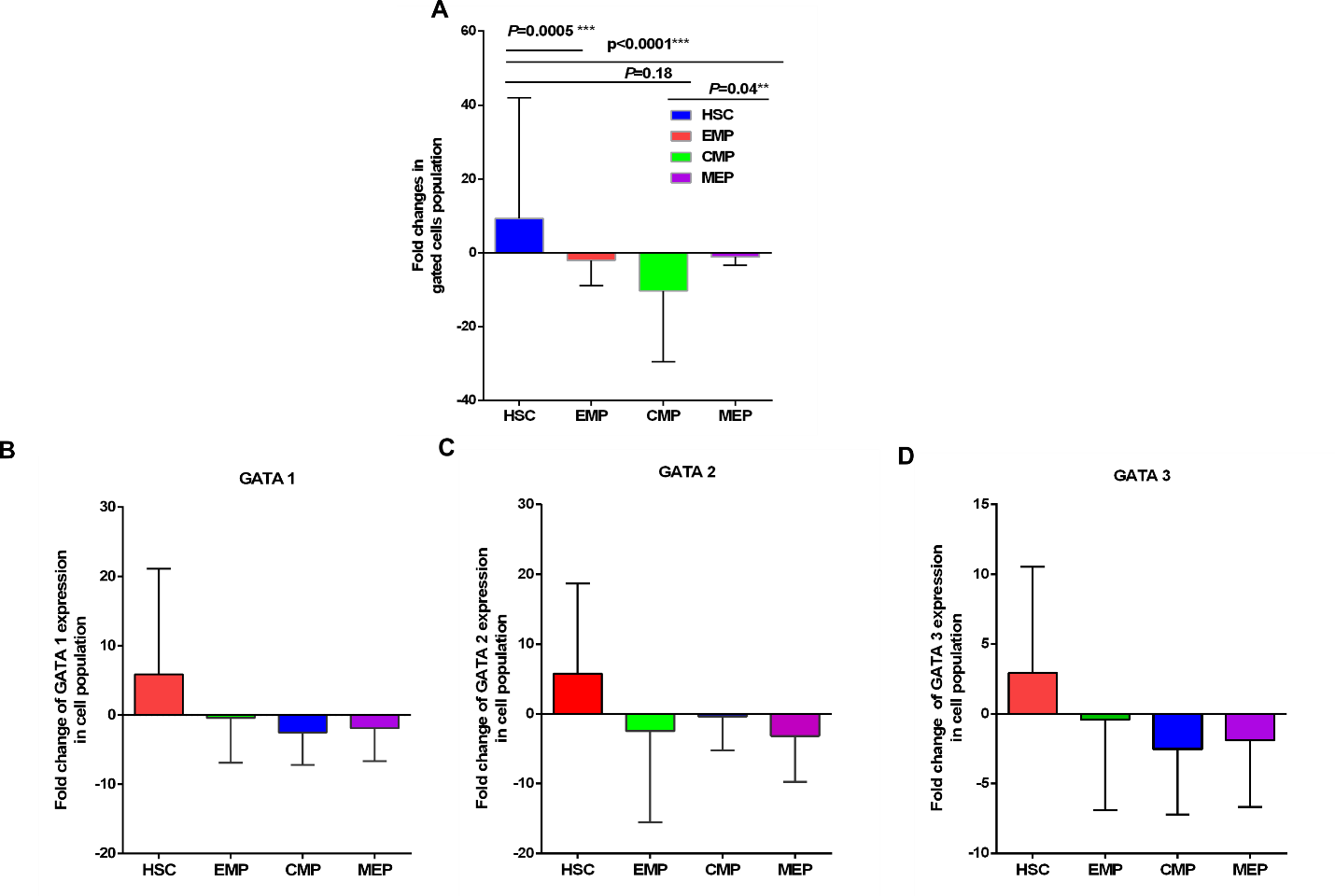


**Figure S2.1. Expression of transcription factor.** **Related to Figure 1 and 2**. GATA transcription factors in overall cells of DENV infected UCB. Hematopoietic Stem Cells were increased, but lineage progenitors were decreased. **Related to Figure 1. (A)** Comparison of different types of cell populations in UCB cells (n=7). Ordinary one-way test ANOVA test was applied. **(B-D)** The cells expressing GATA-1, GATA-2 and GATA-3 were selected respectively to show the comparison against each cell population (n=6). Data were analyzed using ordinary one-way test ANOVA test and shown as mean ± SEM (*P<0.005 ,* *P=0.01)

**
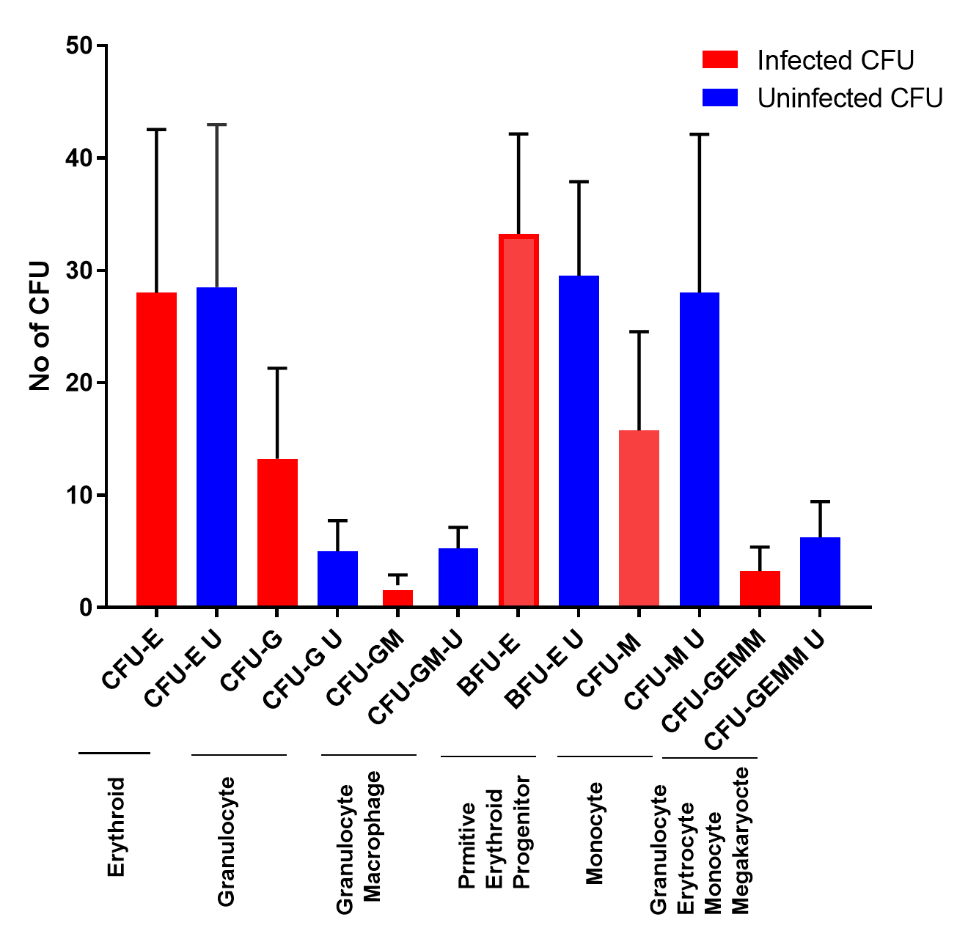
 Supplementray Figure S2.2**

**Figure S2.2**. **Colony Forming Assay. Related to Figure 2.** Thirty-five hundred HSPCs were seeded in semisolid medium to allow colony formation for 2 weeks. The colonies were classified into 6 categories (CFU-E, BFU-E, CFU-G, CFU-M, CFU-GM, CFU-GEMM) and characterized and scored according to their morphology with an inverted microscope (N=4). Myeloid progenitor cells were reduced after DENV infection in colony-forming assay

**Supplementary Figure 3**


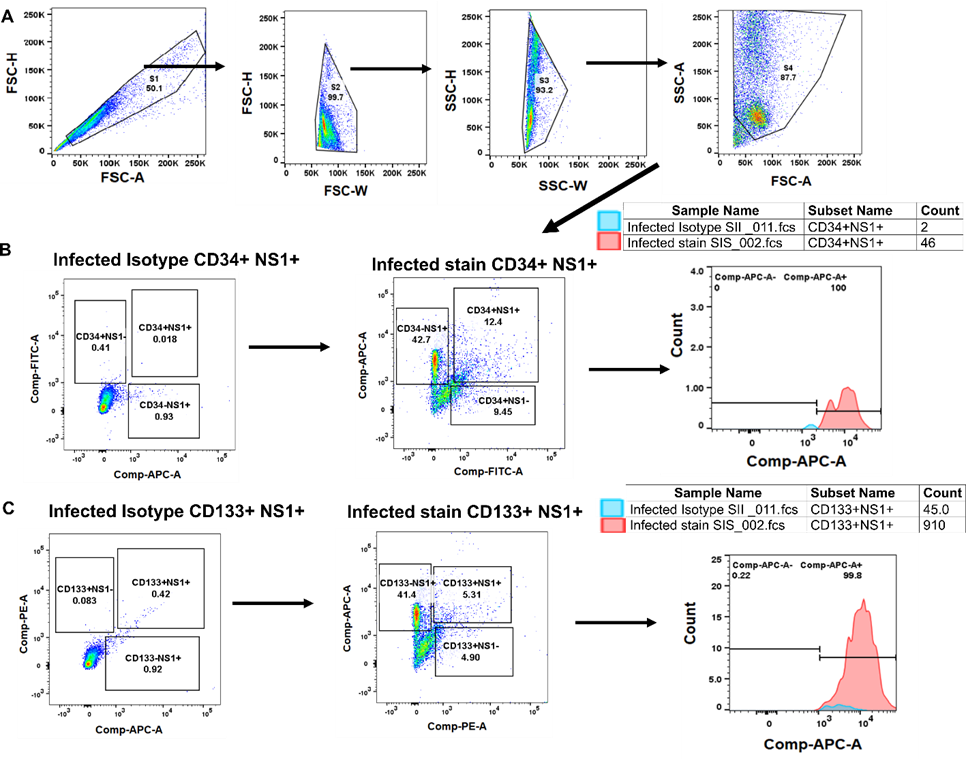
**Supplementary Figure S3.1**

**Figure S3.1: Gating strategy applied to characterize CD34+/-, CD133+/- with NS1+ in DENV infected UCB.** (A). Cells were first gated for singlets for forward and side scatter (FSC-A vs FSC-H, FSC-W vs FSC-H, SSW vs SSSH and whole cell FSC-A and SSC-A and debris were excluded using flow Jo 10. (B-C). Live cells were selected to further gate CD133^+/-^ or CD34^+/-^ with NS1^+/-^ for further analysis. Populations were initially gated in the quadrant plot for isotype. Hereafter, infected population of CD34^+/-^NS1^+/-^ and CD133^+/-^NS1^+/-^ were gated to measure the percentage of cell infected. A histogram was also plotted.

 **Supplementary Figure S3.2**

**Figure S3.2. Statistical analysis of the frequency of NS1^+^ in the gated population of** **CD133^+^CD34^+^. Related to Figure 4.** (A) Frequencies of NS1 on CD133^+^CD34^+^ from the representative donor are presented. Calculated frequencies are presented outside the fluorescent gating plot. Changes of the relative gating percentage of NS1 in the CD133^+^CD34^+^ population were depicted by the color intensity of the fluorescent gating plot using FlowJo. (B) **Statistics to show the percent gated cells of NS1+ on CD34+ CD133+ parent population**. Statistics of NS1 was examined by first gating the relative events of NS1^+^ on the plot of parent population of CD34^+^ CD133^+^ using quadrant gating. Frequency of percent gated events of NS1^+^ was calculated by dividing the gating applied to NS1^+^ on the CD133^+^ CD34^+^ plot by the number of events collected for CD133^+^ CD34^+^.

**Supplementary Figure S4**

**
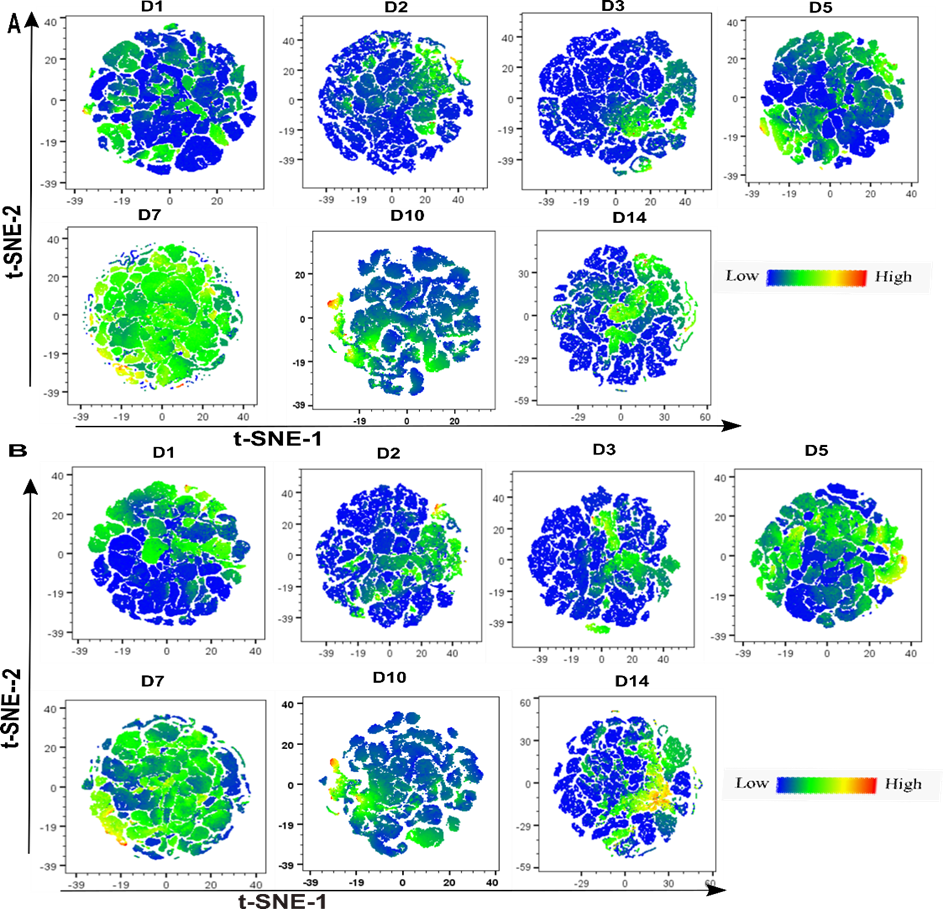
**

**Figure S4. t-SNE to show the CD133^+^CD34 ^+^ NS1^+^ heterogeneity.** **Related to Figure 6.** (A) t-SNE landscape to indicate the distribution of CD133^+^ on CD34^+^ NS1^+^. (B) Distribution of CD34^+^ in CD133^+^NS1^+^ population. Gated all live populations on FSC- A and SSC-A plots were concatenated to show the frequency of predicted CD133+ and CD34+ surface marker expression (Down sample event=50,000).

**
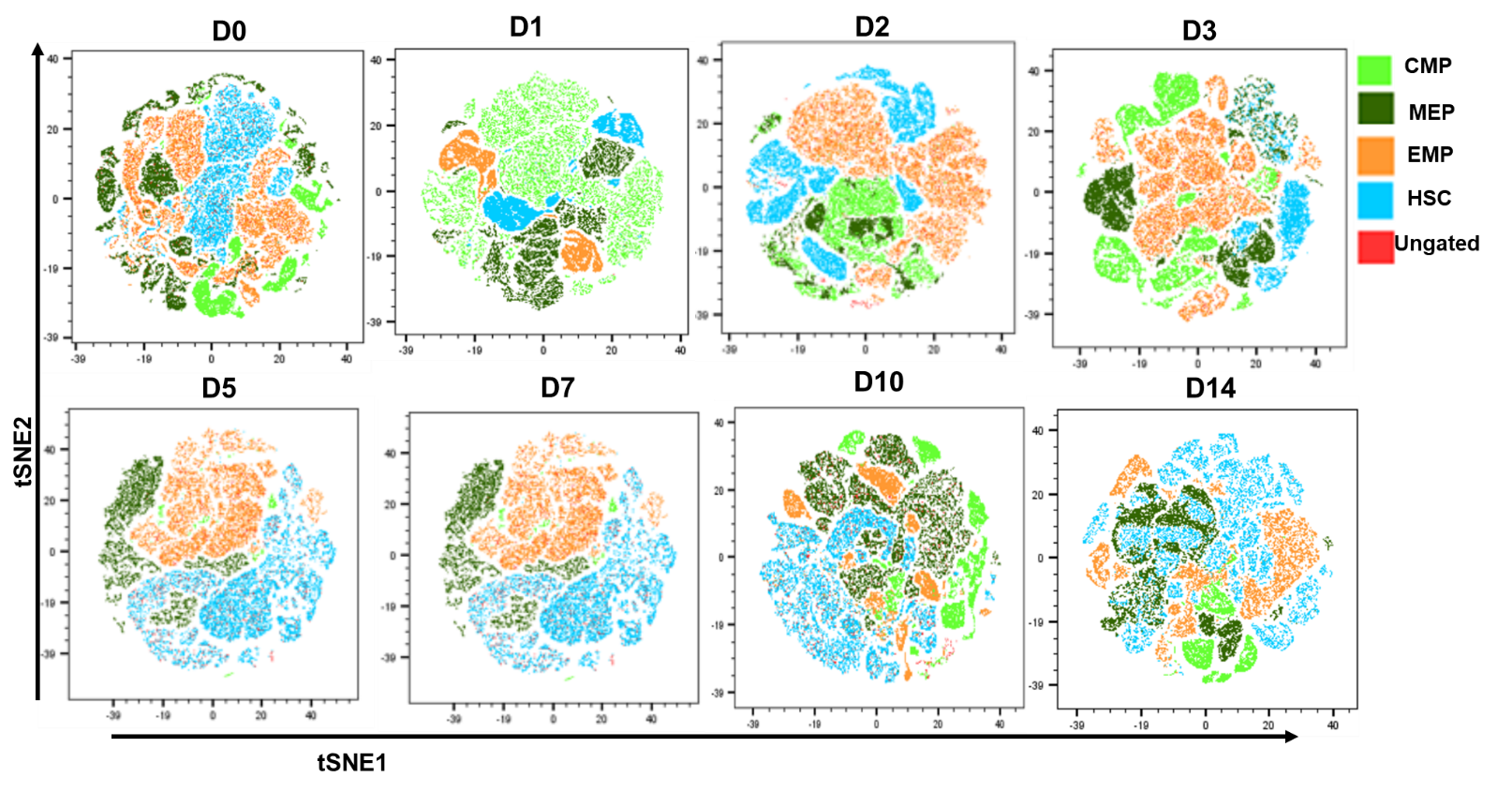
 Supplementary Figure S4.1**

**Figure S4.1. Expression level of specific cell population using t-SNE plot of cell density. Related to figure 6.** Visualization of the defined subpopulation of various lineages using t-SNE in DENV infected UCB. Predicted distribution and frequency of each cell types based on co-expression of various surface markers of stem cells (CD34^+^/CD133^+)^ , Megakaryocytic (CD61^+^CD41^+^), Phagocytic (CD14^+)^ and multipotent progenitor cells expression marker (CD45^+^) with NS1^+^ .


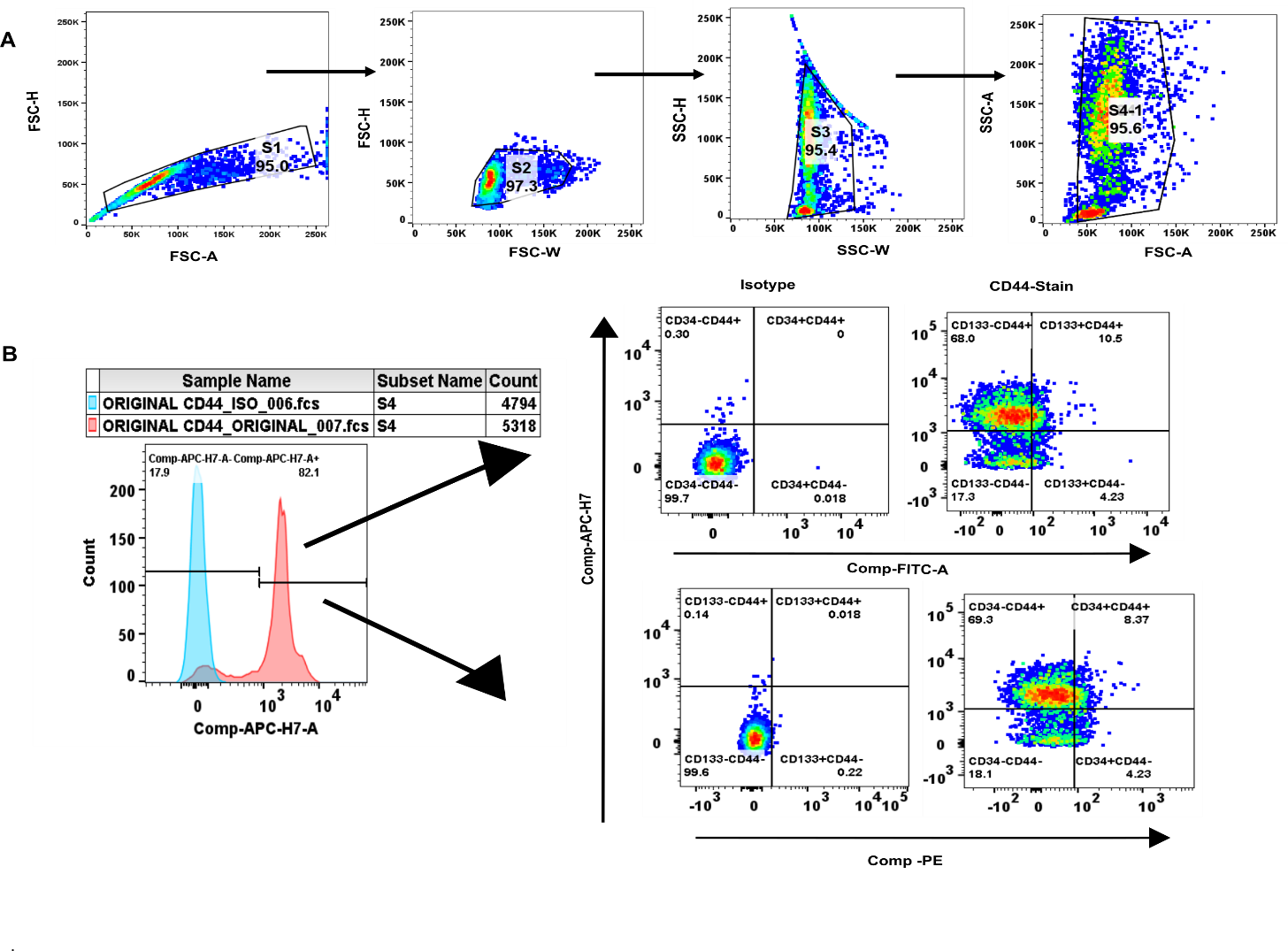
 **Supplementary Figure S5**


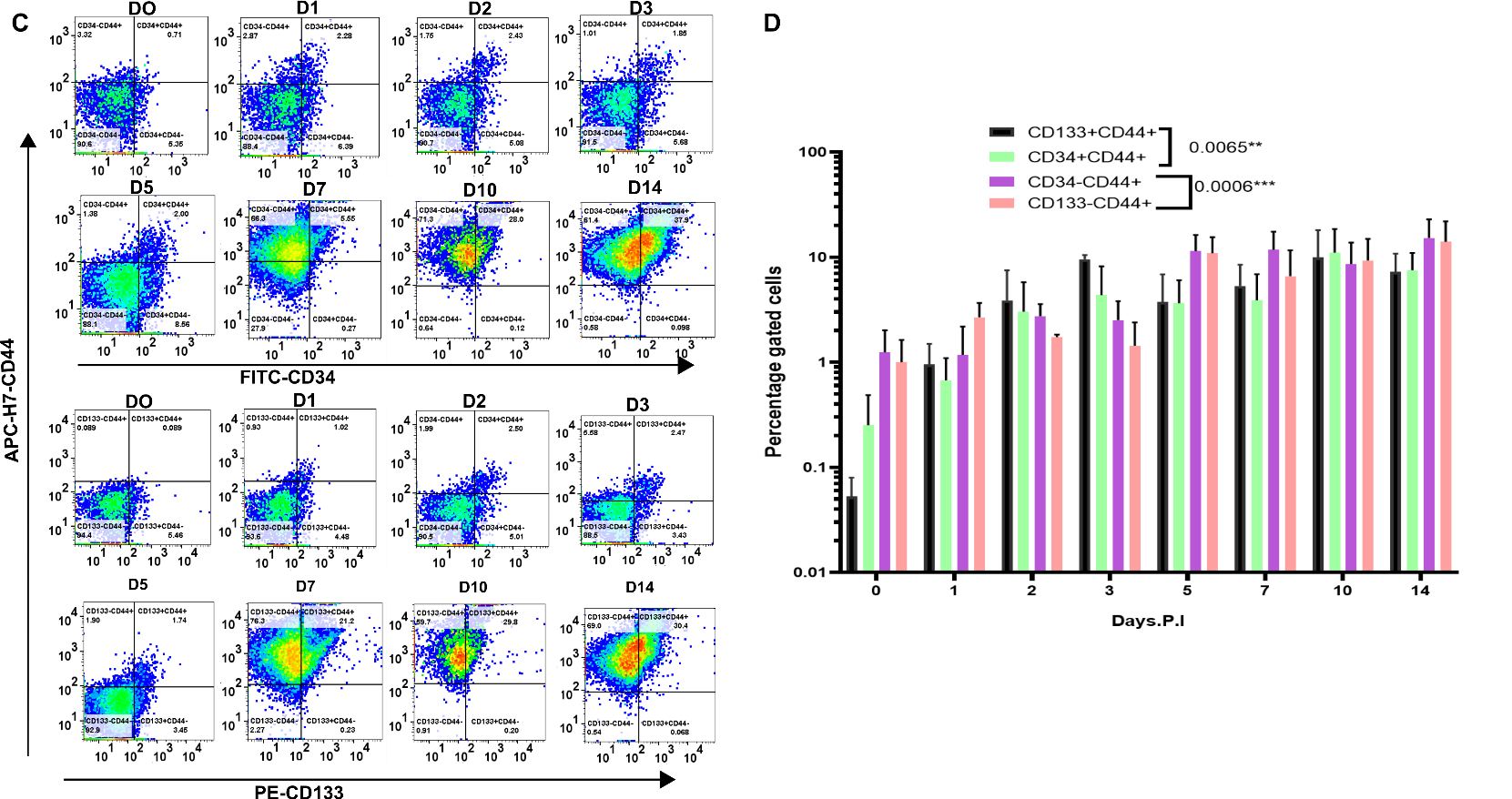


**Figure S5.** (A) Gating strategy applied for enumeration of CD44.Cells were first gated for singlets (FSC-A vs FSC-H, FSC-W vs FSC-H, SSW vs SSSH and whole cell FSC-A and SSC-A.(B) The gate were further analyzed for the enumeration of original CD44 in UCB, taking in only the live cells. The histogram chart of original CD44 and isotype is shown. Additionally, gating percentages of CD34^+^CD44^+^, CD133^+^CD44^+^ subsets and isotypes were determined from this gated population. (C-D) Representative gating panels from one donor. The results are representative of three independent experiment.

**Supplementary Tables**

**Supplementary Table S1, related to Figure 1A. Viral load of respective donors (pfu/ml). D0 (2 h of post infection).**

| **Days PI** | **Donor 1** | **Donor 2** | **Donor 2** | **Donor 4** | **Donor 5** | **Donor 6** | **Donor 7** |
| --- | --- | --- | --- | --- | --- | --- | --- |
| D0 | 0 | 0 | 0 | 0 | 0 | 0 | 0 |
| D1 | 38 | 88 | 25 | 0 | 0 | 188 | 0 |
| D2 | 63 | 38 | 225 | 150 | 150 | 2800 | 88 |
| D3 | 163 | 163 | 550 | 0 | 0 | 15625 | 0 |
| D5 | 100 | 13 | 0 | 488 | 488 | 27125 | 900 |
| D7 | 0 | 0 | 16500 | 2300 | 2300 | 32125 | 0 |
| D10 | 288 | 0 | 12250 | 0 | 0 | 4000 | 0 |
| D14 | 0 | 0 | 88 | 0 | 0 | 0 | 7825 |

**Supplementary Table S2: Average values of actual number of gated cells expressing GATA-1, GATA-2 and GATA-3 obtained after gating only live cells (n=6).**

|  | **GATA1** | | | |
| --- | --- | --- | --- | --- |
| **Days PI** | **HSC** | **EMP** | **CMP** | **MEP** |
| **D0** | -0.06 | 1.10 | 4.21 | -5.36 |
| **D1** | 6.20 | -6.97 | -3.41 | -0.74 |
| **D2** | -8.90 | -27.16 | 4.75 | 1.82 |
| **D3** | 0.19 | -0.23 | 4.66 | 0.59 |
| **D5** | 11.37 | -3.02 | -8.69 | -5.31 |
| **D7** | 10.12 | -9.65 | 0.59 | 1.87 |
| **D10** | 2.90 | 10.14 | -3.22 | -7.90 |
| **D14** | 1.14 | 16.23 | -2.05 | 2.90 |

|  | **GATA2** | | | |
| --- | --- | --- | --- | --- |
| **Days PI** | **HSC** | **EMP** | **CMP** | **MEP** |
| **D0** | -0.59 | -0.50 | 1.38 | -1.82 |
| **D1** | 0.83 | -6.30 | 0.52 | -0.94 |
| **D2** | -0.29 | -13.43 | 2.65 | 1.12 |
| **D3** | 1.48 | -3.89 | 0.73 | -1.04 |
| **D5** | -1.76 | -2.27 | 2.28 | -1.30 |
| **D7** | 6.85 | 3.49 | -0.09 | 2.34 |
| **D10** | -0.69 | 14.27 | -0.96 | -2.11 |
| **D14** | 0.55 | 13.70 | -0.38 | 0.70 |

|  | **GATA3** | | | |
| --- | --- | --- | --- | --- |
| **Days PI** | **HSC** | **EMP** | **CMP** | **MEP** |
| **D0** | 2.670833 | 5.133333 | 2.555 | -0.99167 |
| **D1** | 7.023333 | 1.503333 | -8.00167 | 1.218333 |
| **D2** | -5.05083 | -14.2883 | 1.221667 | -0.48667 |
| **D3** | -1.6175 | -0.23167 | 2.098333 | 0.625 |
| **D5** | 7.525 | -4.28833 | -10.305 | -3.56167 |
| **D7** | 1.035 | 4.18 | -2.63167 | -0.285 |
| **D10** | 1.5475 | -0.53833 | -3.435 | -6.35333 |
| **D14** | -1.4975 | 5.078333 | -1.70667 | 2.033333 |

**Supplementary Table S3: Actual number of CD133+/-, CD34+/ cell population from DENV infected UCB obtained after gating cell only live cells and analyzed using Flow jo10**

|  |  |  |  |  | |  |  |
| --- | --- | --- | --- | --- | --- | --- | --- |
| **Days PI** | **Donor 1** | **Donor 2** | **Donor 3** | **Donor 4** | **Donor 5** | **Donor 6** | **Donor 7** |
| **0** | 0.3 | 0.88 | 0.74 | 0.084 | 29.3 | 3.6 | 0.41 |
| **1** | 0.7 | 0.79 | 0.94 | 0.18 | 59.5 | 0.27 | 1.59 |
| **2** | 0.6 | 0.93 | 1.17 | 0.12 | 49.1 | 0.54 | 4.08 |
| **3** | 0.59 | 0.74 | 1.23 | 0.58 | 85.2 | 0.52 | 1.38 |
| **5** | 0.88 | 0.79 | 2.12 | 1.08 | 26.2 | 12.6 | 1.11 |
| **7** | 1.19 | 1.21 | 2.5 | 1.84 | 13.4 | 0.87 | 1.29 |
| **10** | 1.78 | 2.8 | 2.08 | 1.2 | 1.04 | 3.72 | 0.37 |
| **14** | 1.23 | 5.3 | 3.38 | 2.44 | 4.27 | 3.12 | 0.68 |

**B**

**A**

**CD133^-^CD34^+^**

|  |  |  | **CD133+CD34+** |  | |  |  |
| --- | --- | --- | --- | --- | --- | --- | --- |
| **Days PI** | **Donor 1** | **Donor 2** | **Donor 3** | **Donor 4** | **Donor 5** | **Donor 6** | **Donor 7** |
| **0** | 0.13 | 0.69 | 0.5 | 0.46 | 26 | 4.4 | 0.42 |
| **1** | 0.53 | 0.79 | 0.79 | 0.025 | 34.5 | 0.18 | 0.69 |
| **2** | 0.49 | 1.14 | 1.3 | 0.043 | 31.4 | 0.65 | 1.36 |
| **3** | 1.22 | 1.6 | 1.9 | 0.76 | 15.2 | 1.4 | 4.23 |
| **5** | 3.08 | 2.12 | 1.76 | 1.27 | 2.12 | 6.07 | 4.46 |
| **7** | 2.09 | 3.08 | 2.75 | 3.1 | 3.28 | 1.47 | 3.32 |
| **10** | 2.93 | 3.53 | 6.79 | 5.17 | 3.91 | 5.95 | 0.015 |
| **14** | 4.18 | 20.3 | 12 | 15.3 | 15.1 | 4.22 | 2.79 |

|  |  |  | **CD133^+^CD34^-^** | |  |  |  |
| --- | --- | --- | --- | --- | --- | --- | --- |
| **Days PI** | **Donor 1** | **Donor 2** | **Donor 3** | **Donor 4** | **Donor 5** | **Donor 6** | **Donor 7** |
| **0** | 0.3 | 0.71 | 0.38 | 0.69 | 1.6 | 0.49 | 0.69 |
| **1** | 0.38 | 2.89 | 0.54 | 2.22 | 0.04 | 0.62 | 1.1 |
| **2** | 0.74 | 1.34 | 1.25 | 2.78 | 0.34 | 0.39 | 3.56 |
| **3** | 1.96 | 1.68 | 1.68 | 2.52 | 0.13 | 0.72 | 2.87 |
| **5** | 5.9 | 1.65 | 1.67 | 1.22 | 0.05 | 1.65 | 2.17 |
| **7** | 1.96 | 1.18 | 2.27 | 5 | 0.31 | 1.04 | 2.1 |
| **10** | 2.53 | 2.46 | 9.74 | 14.2 | 1.54 | 2.95 | 5.33 |
| **14** | 13.3 | 8 | 13 | 15 | 1.15 | 3.6 | 4.21 |

**C**

**Supplementary Table S4: Actual number of CD133+ NS+, CD34+NS1+ and CD133+CD34+NS1+ cell population from DENV infected UCB obtained after gating only live cells and analyzed using Flow jo10**

**CD133+NS1+**

| **Days PI** | **Donor 1** | **Donor 2** | **Donor 3** | **Donor 4** | **Donor 5** | **Donor 6** | **Donor 7** |
| --- | --- | --- | --- | --- | --- | --- | --- |
| **0** | 0.54 | 11.4 | 2.4 | 19.3 | 14.8 | 4.33 | 12.2 |
| **1** | 0.27 | 5.21 | 4.3 | 45.5 | 6.61 | 0.16 | 12.4 |
| **2** | 0.24 | 5.25 | 6.96 | 26.6 | 17.4 | 0.88 | 17.8 |
| **3** | 0.68 | 28 | 12.5 | 14.5 | 2.57 | 1.7 | 11.9 |
| **5** | 2.52 | 13.3 | 10.8 | 2.17 | 7.02 | 5.52 | 13.4 |
| **7** | 0.38 | 14 | 0.72 | 3.21 | 8.94 | 1.1 | 9.74 |
| **10** | 4.97 | 12.9 | 14.3 | 3.15 | 4.75 | 4.08 | 31.6 |
| **14** | 3.31 | 16.2 | 8.14 | 14.4 | 48.5 | 3.6 | 67 |

**A**

|  |  |  |  |  | |  |  |
| --- | --- | --- | --- | --- | --- | --- | --- |
| **Days PI** | **Donor 1** | **Donor 2** | **Donor 3** | **Donor 4** | **Donor 5** | **Donor 6** | **Donor 7** |
| **0** | 3.5 | 0.69 | 10.1 | 10.3 | 2.8 | 1.35 | 49.7 |
| **1** | 22.2 | 0.11 | 8.58 | 4.79 | 2.5 | 0.14 | 45.5 |
| **2** | 24.6 | 0.25 | 15.8 | 4.87 | 7.73 | 0.43 | 26.4 |
| **3** | 15.7 | 0.86 | 1.85 | 19.5 | 5.95 | 2.04 | 9.49 |
| **5** | 15.8 | 3.11 | 7.21 | 11.2 | 5.51 | 8.4 | 1.88 |
| **7** | 10.7 | 1.43 | 14 | 12.5 | 0.45 | 1.63 | 3.04 |
| **10** | 33.8 | 5.05 | 6.6 | 10.6 | 16.2 | 6.97 | 3.33 |
| **14** | 71.7 | 5.15 | 39.9 | 15.3 | 6.94 | 4.23 | 12.3 |

**B**

**CD34+NS1+**

| **C** |  |  |  | | **CD34+CD133+NS1+** |  |  |
| --- | --- | --- | --- | --- | --- | --- | --- |
| **Days PI** | **Donor 1** | **Donor 2** | **Donor 3** | **Donor 4** | **Donor 5** | **Donor 6** | **Donor 7** |
| **0** | 29.3 | 3.08 | 36 | 1.05 | 5.7 | 10.1 | 67 |
| **1** | 9.95 | 1.61 | 37 | 0.85 | 10.25 | 11.2 | 59 |
| **2** | 5.5 | 3.46 | 11.0 | 5.7 | 5.3 | 9.11 | 82 |
| **3** | 8.3 | 21 | 24.08 | 6.04 | 8.17 | 4.2 | 21.04 |
| **5** | 7.2 | 8.37 | 11.4 | 12.6 | 28.2 | 7.6 | 37 |
| **7** | 6.3 | 21 | 14.2 | 13.6 | 29.4 | 7.3 | 37 |
| **10** | 2.69 | 2.791 | 5.38 | 6.68 | 21.7 | 2.4 | 14.7 |
| **14** | 2.29 | 46 | 22.11 | 3.7 | 7.9 | 8.8 | 25.67 |

**Supplementary Table S5: Serological confirmation anti-DENV IgG and IgM antibodies in UCB serum, detected by conducting on-site Duo Dengue CTK Biotech Ag IgG/IgM rapid test.**

| **Mother** | **Age** |  | **Serological confirmation** | |  | **Dengue clinical symptoms** |
| --- | --- | --- | --- | --- | --- | --- |
|  |  |  | **IgG** | **IgM** |  |  |
| 1 | 39 |  | - | - |  | NCS |
| 2 | 31 |  | - | - |  | NCS |
| 3 | 32 |  | - | - |  | NCS |
| 4 | 32 |  | - | - |  | NCS |
| 5 | 35 |  | - | - |  | NCS |
| 6 | 36 |  | - | - |  | NCS |
| 7 | 37 |  | - | - |  | NCS |
| 8 | 33 |  | - | - |  | NCS |
| 9 | 31 |  | - | - |  | NCS |
| 10 | 33 |  | - | - |  | NCS |
| 11 | 36 |  | - | - |  | NCS |
| 12 | 33 |  | - | - |  | NCS |
| 13 | 35 |  | - | - |  | NCS |
| 14 | 35 |  | - | - |  | NCS |

**Abbreviation: NCS:** No clinical symptoms.

| **Experimental Panel** | **Name** | **Source** | **Clone** | **Identifier** |
| --- | --- | --- | --- | --- |
| **HSC** | CD133-PE  CD34-FITC | Biolgend  BD Biosciences | 29C3  581 | Cat#372804  Cat#555821 |
| **Myeloid Cell Lineage** | CD45RA-APC-H7  CD61-Per-Cp-Cy5.5  CD14-500  CD41a-V450 | BD Biosciences  eBiosciences | 5H9  VI-PL2  M5E2  HIP8 | Cat#561212  Cat#564173  Cat#581391  REF#480419-42 |
| **GATA-Panel** | CD133-PE-vio770  CD34-BV-421  CD61-BV-510  CD45-BUV395  CD41-Per-Cp-Cy5.5  CD14-BUV-737  GATA-1-PE  GATA-2-FITC  GATA-3-APC | Miltenyi Biotech  BD  BD  BD  BD  BD  Research & Design | VI-PL2  HI30(RUO)  HIP8(GMP)  M5E2 | 130-113-750  Cat#562577  Cat#563303  Cat#563792  Cat#340931  Cat#612763  IC2046F  IC1779P  IC63301A-025 |
| **Isotype Control** | BV421 IgG1  BV510 IgG1  BUV395 IgG1  Per-Cp-Cy5.5 IgG1  BUV 737 IgG2a  PE-Rat IgG2b  Flourescein Goat IgG | BD Biosciences  Research & Design | X40  X40  HI30  HIP8  M5E2 | Cat#562438  Cat#562946  Cat# 563547  Cat# 552834  Cat# 612765  IC013P  IC108F |

**Supplementary Table S6A: Phenotypic Panel of antibodies for Flow cytometry of stem cell mediated antibodies and GATA Transcription factors**

**Supplementary Table S6B: Cell surface phenotypes of various hematopoietic stem and progenitor cell population to isolate HSPC in NS1+ stained DENV infected UCB.**

| **Marker Phenotype** | **Cell Type** | **Source** | **Clone** | **Identifier** |
| --- | --- | --- | --- | --- |
| **CD133-PE** | HSC/ Hematopoietic stem and Progenitor Cells | Biolgend | clone 7 | Cat#372804 |
| **CD34-FITC** |  | BD Pharmingen ^TM^ | 581 | Cat#555821 |
| **CD45RA APC-H7** | HSC/Multipotent progenitor cells | BD Biosciences | 5H9 | Cat#561212 |
| **CD41a- V450** | Platelets precursor MEP, EMP | BD Pharmingen^TM^ | HIP8 | Cat #561425 |
| **CD61 Per-Cp-Cy5.5** | Platelets precursor MEP, EMP | BD Biosciences | VI-PL2 | Cat#564173 |
| **CD14 V500** | Precursor for monocyte, can differentiate into macrophage and myeloid lineage | BD Horizon | M5E2 | Cat#561391 |
| **NS1** | Dengue Virus Non -structural protein | Thermo Fisher scientific | Alexa ^TM^  flour 647 | A30009 Labelling kit |
|  |  |  |  |  |
| **Isotype Control** | FITC-IgG1k | BD Pharmingen^TM^ | MOPC-21 | Cat#555748 |
|  | PE-IgG1 k |  | MOPC-21 | Cat#555749 |
|  | Mouse APC-H7 IgG2a k |  | GI55-178 | Cat#560897 |
|  | Mouse PerCP-Cy ^TM^ 5.5 IgG1 k |  | MOPC-21 | Cat#564173 |
|  | Mouse V450 IgG2a k |  | GI55-178 | Cat#560550 |
|  | Mouse V500 IgG2a k |  | GI55-178 | Cat#561221 |
|  | AF-647Mouse IgG2a k |  | G155-178 | Cat#557715 |
